# Injectable Granular Hydrogels Enable Avidity-Controlled Biotherapeutic Delivery

**DOI:** 10.1021/acsbiomaterials.3c01906

**Published:** 2024-02-15

**Authors:** Arielle M. D’Elia, Olivia L. Jones, Gabriela Canziani, Biplab Sarkar, Irwin Chaiken, Christopher B. Rodell

**Affiliations:** † School of Biomedical Engineering, Science and Health Systems, Drexel University, Philadelphia, Pennsylvania 19104, United States; ‡ Department of Biochemistry and Molecular Biology, Drexel University College of Medicine, Philadelphia, Pennsylvania 19102, United States

**Keywords:** hydrogel, sustained release, cyclodextrin, avidity, bioconjugation

## Abstract

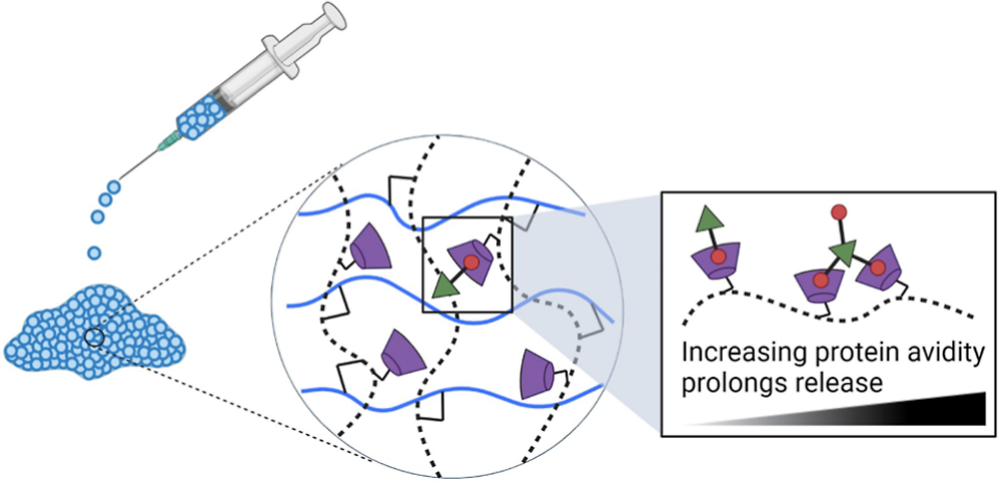

Protein therapeutics represent a rapidly growing class of pharmaceutical agents that hold great promise for the treatment of various diseases such as cancer and autoimmune dysfunction. Conventional systemic delivery approaches, however, result in off-target drug exposure and a short therapeutic half-life, highlighting the need for more localized and controlled delivery. We have developed an affinity-based protein delivery system that uses guest–host complexation between β-cyclodextrin (CD, host) and adamantane (Ad, guest) to enable sustained localized biomolecule presentation. Hydrogels were formed by the copolymerization of methacrylated CD and methacrylated dextran. Extrusion fragmentation of bulk hydrogels yielded shear-thinning and self-healing granular hydrogels (particle diameter = 32.4 ± 16.4 μm) suitable for minimally invasive delivery and with a high host capacity for the retention of guest-modified proteins. Bovine serum albumin (BSA) was controllably conjugated to Ad via EDC chemistry without affecting the affinity of the Ad moiety for CD (*K*_D_ = 12.0 ± 1.81 μM; isothermal titration calorimetry). The avidity of Ad–BSA conjugates was directly tunable through the number of guest groups attached, resulting in a fourfold increase in the complex half-life (*t*_1/2_ = 5.07 ± 1.23 h, surface plasmon resonance) that enabled a fivefold reduction in protein release at 28 days. Furthermore, we demonstrated that the conjugation of Ad to immunomodulatory cytokines (IL-4, IL-10, and IFNγ) did not detrimentally affect cytokine bioactivity and enabled their sustained release. Our strategy of avidity-controlled delivery of protein-based therapeutics is a promising approach for the sustained local presentation of protein therapeutics and can be applied to numerous biomedical applications.

## Introduction

Protein therapeutics have become a prime market force,^1^ as they exhibit high specificity and efficacy in targeting disease pathways.^2^ Due to their remarkable diversity and biological versatility, protein therapeutics are used to treat a wide range of disorders, including autoimmune diseases, cancer, metabolic disorders, and rare genetic conditions.^3^ Moreover, growth factors and cytokines are widely employed in regenerative medicine applications to promote tissue growth repair.^4−8^ While biotherapeutics are commonly delivered systemically, their sensitivity to proteasomal degradation and relatively short systemic half-lives lead to a need for repetitive dosing to maintain durable therapeutic effects. Additional barriers to clinical use include potential systemic off-target effects such as toxicity or systemic (rather than local) modulation of the immune response.^9−11^ Hence, there is a critical need for controlled release systems that can concentrate drug effects at the site of action and maintain a long-term therapeutic response. When used as drug carriers, hydrogels can encapsulate therapeutic agents and sustain their release to enable precise dosing, reduce administration frequency, and minimize systemic off-target side effects.^12^ However, since the release mechanisms are often purely diffusive, these systems frequently exhibit rapid burst release.^13^

Affinity-based drug delivery systems can enable sustained delivery while minimizing burst release, as they use specific interactions between the biomaterial carrier and target molecules to allow for precise and controlled drug release at a desired site.^14^ The use of noncovalent and reversible supramolecular interactions within these systems allows for the tuning of drug release kinetics.^15^ For example, guest–host (GH) interactions involve the reversible binding of a guest molecule within the hydrophobic cavity of a host molecule, allowing for the modulation of drug release behavior by reducing drug diffusivity, resulting in extended drug release.^16−24^ Specifically, the host macrocycle β-cyclodextrin (CD) is widely used due to its biocompatibility, excellent water solubility, and capability to host a diverse range of guest molecules within its binding cavity, primarily through hydrophobic interactions.^25−28^ Notably, the GH complex between CD and adamantane (Ad) exhibits a relatively high affinity (*K*_D_ = 10 μM),^29^ and the two readily bind under aqueous conditions.^25^ Molecular guests, including Ad, have been used as a linking moiety to enhance the retention of drug cargo, facilitating sustained release.^30,31^ By engaging multiple GH interactions simultaneously, avidity (i.e., the effective interaction of multiple noncovalent bonds) can be leveraged to further improve bond strength,^32,33^ which provides a unique opportunity for modulating therapeutic release through valency of the guest or host groups.

Granular hydrogels have emerged as promising materials for tissue engineering that have the potential to be used for local drug delivery such as by harnessing the inclusion of GH complexes. These nonhomogenous hydrogels consist of physically jammed particulates (i.e., granules of a covalently cross-linked hydrogel), allowing for a highly interconnected porous structure that supports cell infiltration.^34^ Importantly, granular hydrogels are desirable for local delivery applications as they are shear-thinning and self-healing, which enables their injection. Furthermore, they possess a highly interconnected porous structure that allows for cell and tissue infiltration.^35,36^ The composition or structure of the microgels can be tuned, so as to control the physical properties of the hydrogel,^37−39^ cell–material interactions,^40−44^ or growth factor delivery.^45,46^ Many applications have focused on the capacity of these materials to modulate cell response, and Phelps et al. have investigated the functionalization of microgels with GH pairs to develop granular hydrogels as a unique microenvironment for cell delivery.^47−49^ The use of supramolecular interactions between such hydrogels and appropriately modified therapeutic cargo may also be used to enhance drug function, including for immune modulation, but these applications remain unexplored.

Herein, we harness the ability to control granular hydrogel properties to create an avidity-based protein delivery system that uniquely leverages multiple GH interactions between CD and Ad to enable the controlled release of guest-modified proteins. To achieve this, we report the preparation of a granular hydrogel composed of copolymerized methacrylated CD (MeCD) and methacrylated dextran (DexMA) that allows for injectable delivery. We furthermore conjugated Ad to proteins using EDC chemistry, allowing for their controlled modification while retaining both the affinity of GH pairs and the activity of biotherapeutics. By incorporating these guest-modified proteins within our granular hydrogels with a high host-capacity, we were able to control the retention and release of proteins through reversible GH binding, with a dependence on valency of the interaction. This tunable valency increased the effective bonding interaction and the statistical likelihood for rebinding, therefore, changing hydrogel avidity. This avidity enabled the retention and sustained release of both a model protein (bovine serum albumin, BSA) and cytokines, yielding a novel strategy for the local and sustained delivery of proteins.

## Experimental Section

### General Materials and Methods

Dextran (MW = 75 kDa) was purchased from Thermo Fisher Scientific, dialysis membranes from Spectrum, and recombinant cytokines from PeproTech. For ITC and SPR samples, phosphate-buffered saline (PBS; Roche Diagnostics GmbH, Mannheim, Germany); sterile dimethyl sulfoxide (DMSO) USP >99.9% (Stemsol, Protide Pharmaceuticals Inc., IL, USA); C1 carboxymethylated, matrix-free chip series S (BR100535); and the Amine Coupling Kit (BR100050) were obtained from Cytiva. Other solvents and general reagents were purchased from TCI America or Sigma-Aldrich and used as received unless otherwise indicated. ^1^H NMR spectra were acquired at 500 MHz (Varian Unity Inova), and chemical shifts are reported relative to the residual solvent peak.

### Synthesis of DexMA and MeCD

CD and dextran were methacrylated via esterification with glycidyl methacrylate (GMA) using a modified version of reported protocols.^50,51^ Briefly, a round-bottom flask was charged with CD or dextran (0.5 g, 1 equiv) and 4-dimethylaminopyridine (50 mg, 0.15 equiv). Under anhydrous conditions, DMSO (15 mL) was added via cannulation followed by the addition of GMA (0.25, 0.5, 1, or 2 equiv). The reaction was allowed to proceed under nitrogen (24 h, 45 °C). MeCD was precipitated from a 10-fold excess of ice-cold acetone and redissolved in deionized (DI) water prior to dialysis for 48 h against DI water (0.1–0.5 kDa MWCO). Dextran was purified by dialysis for 5 days against DI water (6–8 kDa MWCO). Products were frozen at −80 °C and lyophilized to yield MeCD and DexMA, respectively, which were stored under nitrogen at −20 °C until further use. For MeCD, the degree of substitution (DS) was determined from ^1^H NMR in DMSO-*d*_6_ as the ratio of the methyl peak (ca. 1.9 ppm) relative to the position 1 anomeric proton (ca. 4.8 ppm). The DS for DexMA was similarly assessed from spectra acquired in D_2_O.

### Hydrogel Formation and Rheological Characterization

Hydrogels were prepared from solutions of DexMA and/or MeCD in PBS containing 5 mM lithium phenyl-2,4,6-trimethylbenzoyl phosphonate (LAP, photoinitiator) by photopolymerization (OmniCure S1500 lamp, λ = 320–390 nm, 10 mW/cm^2^). Polymerization of 1.25, 2.5, 5, and 10%_w/v_ DexMA and/or MeCD was monitored in real time by oscillatory shear rheology (Discovery HR20, TA Instruments) using a photocuring stage and a 20 mm sandblasted stainless-steel plate top geometry (200 μm gap). After a prepolymerization period (2 min), samples were irradiated (5 min) concurrent with oscillatory time sweeps (1.0 Hz, 1.0% strain). Frequency sweeps (0.1–100 Hz, 1.0% strain) and strain sweeps (1.0 Hz, 0.01–1000% strain) were subsequently conducted to ensure that assessments were conducted within the linear viscoelastic region.

### Granular Hydrogel Formulation and Characterization

Microgels were formed by extrusion fragmentation (EF). A 1 mL precursor solution of either DexMA (5%_w/v_) or DexMA + MeCD (5%_w/v_ and 10%_w/v_, respectively) was photo-cross-linked in a 3 mL syringe (10 mW/cm^2^, 5 min) after calibration under a partial syringe to account for light attenuation. The syringe was rotated halfway through the curing process. The bulk hydrogel was subsequently extruded 10 times each through 18G, 20G, 22G, and 25G emulsifying needles (Scientific Commodities) and finally through a 30G needle (McMaster-Carr). After fragmentation, microgel suspensions were centrifuged (2 min, 2000 rcf) to induce particle jamming, and the supernatant was decanted; this step was repeated 3 times.

Particle size was quantified by the inclusion of fluorescein *O*-methacrylate (1 mM) during photopolymerization. Microgel samples (100 μL) were collected after each stage of the EF process, diluted 10-fold by PBS, and imaged at 10x by fluorescence microscopy (Leica, DMI 6000B). For quantification (ImageJ), images were background subtracted, and the microgel diameter was measured; for each particle, four diameter lines were manually drawn and averaged to obtain the microgel diameter. Built-in ImageJ functions were used to determine the circularity and aspect ratio. Microgel morphology was analyzed for 200 microgels per group.

Rheological properties of the granular hydrogel were assessed using a parallel-plate geometry (20 mm, 1 mm gap) via oscillatory time sweeps (1.0 Hz, 1.0% strain), oscillatory frequency sweeps (0.01–100 Hz, 1.0% strain), oscillatory strain sweeps (1.0 Hz, 0.01–1000% strain), and continuous flow experiments, with the shear rate linearly ramped from 0.005 to 50 s^–1^ and returned. Shear-thinning and recovery experiments were performed at periodically applied 500% (high, 1 min) and 0.5% (low, 2 min) strains, each at 1.0 Hz.

### Bovine Serum Albumin and Cytokine Bioconjugation

BSA (Tocris Bioscience) was dissolved in MES buffer (50 mM, pH = 5.8) at a concentration of 5 mg/mL. Subsequently, 6-aminofluorescein (FAM) was added (1.25, 2.5, 5, or 10 equiv to BSA) followed by a 10-fold molar excess of fresh 1-ethyl-3-(3-(dimethylamino)propyl)carbodiimide (EDC), relative to FAM. The reaction was carried out for 2 h at room temperature, and the product (FAM–BSA) was purified by size exclusion chromatography (PD-10, Cytiva). Fractions were concentrated by centrifugal filtration (10 kDa MWCO), washed with DI water, and lyophilized. BSA concentration was determined via absorbance (Protein A280, NanoDrop One C; Thermo Fisher Scientific), and the extent of BSA modification by FAM was assessed by fluorescence intensity, relative to standard curves (BioTek Synergy H1 microplate reader, λ_ex/em_ = 480/525 nm).

Heterobifunctional aminated PEG-Ad (5 kDa, Ruixibio) was conjugated to BSA, recombinant murine interleukin 10 (IL-10), interleukin 4 (IL-4), and interferon γ (IFNγ) using identical reaction conditions and purification, sparing size exclusion chromatography. After cytokine conjugate formation (Ad-IL-10, Ad-IL-4, and Ad-IFNγ), BSA was included (0.1%_w/v_) as a carrier protein prior to purification by dialysis against Milli-Q water (3.5–5 kDa MWCO, 48 h) and lyophilization. Control cytokines underwent identical processing without EDC inclusion.

To generate BSA modified by both Ad and fluorescent labels to monitor in vitro release, sequential modifications were performed. For FAM–BSA–Ad, FAM was conjugated (5 equiv), followed by aminated PEG–Ad (1.25, 2.5, 5, or 10 equiv). Conjugation of additional fluorophores to Ad–BSA products (0, 2.5, and 5 equiv of Ad) for concurrent release studies was performed by reacting Ad–BSA with the succinimidyl esters of Pacific Blue, Fluorescein, and Alexa Fluor 555 (Thermo Fisher Scientific), respectively. Briefly, Ad–BSA (5 mg/mL in 0.1 M sodium bicarbonate buffer, pH 8.5) was reacted with a fivefold molar excess of fluorophore for 4 h at room temperature prior to purification as described above.

### Isothermal Titration Calorimetry

The thermodynamic dissociation constants (*K*_D_) of CD to Ad and four Ad–BSA conjugates were determined at 25 °C using a high-precision VP-ITC calorimeter (MicroCal, Malvern Panalytical). To perform the titration of CD against a constant concentration of free or BSA-conjugated Ad, 1 mM CD was added stepwise (4 μL aliquots, 8 s) into the reaction cell (1.4 mL) containing 10 μM Ad or Ad–BSA. The heat associated with CD binding to Ad was obtained by subtracting the heat of dilution from the heat of reaction. The resulting heat changes were integrated over a time range of 240 s, and the obtained values were fit to a standard single site binding model using Origin 7.0 software. Data from three independent replicates were fit simultaneously for each Ad–BSA conjugate.

### Surface Plasmon Resonance

Surface plasmon resonance (SPR) experiments were performed on a Biacore S200 biosensor (Cytiva) at 25 °C using PBS-P (10 mM Phosphate, 150 mM NaCl, pH 7.4, 0.005% P-20 and 1% DMSO) as both the running and sample buffers. The C1 sensor chip was docked and tested for BSA adsorption and then derivatized by coupling of the ligand 6-(6-aminohexyl)amino-6-deoxy-β-cyclodextrin, prepared as previously described.^21^ The coupling conditions were scouted as a function of the ligand concentration at pH 7.4 by injecting CD in PBS-P at 10, 50, 250, and 1250 μM. Using 10 min injections of freshly mixed 1:1 50 mM *N*-hydroxy-succinimide (NHS) and 200 mM EDC to activate carboxyl groups followed by 10 min injections of 50 or 250 nM ligand injections, 20, 100, and 180 RU CD were coupled on flow cells 2 (FC2), 3 (FC3), and 4 (FC4), respectively. Following the activation of flow cell 1 (FC1), a second activation (5 min) pulse of fresh 1:1 NHS/EDC was implemented to couple BSA by injecting 100 μM in PBS at pH 7.4 and minimize injected BSA, or Ad–BSA, adsorption to the reference sensor. In the same way, FC4 with CD was activated a second time to couple BSA. The proportion of BSA coupled with CD to this sensor surface was 1:2 (90 RU BSA and 180 RU CD) to abrogate unmodified BSA adsorption. The specificity of the CD–Ad interaction was confirmed when no net binding was detected after injecting control samples of PBS-P buffer with 100 μM unmodified BSA. Direct binding was determined by injecting duplicate BSA or Ad–BSA at 6.25, 12.5, 25, 50, and 100 μM over all flow cells at a flow rate of 20 μL/min using a single cycle kinetic titration mode where associations were monitored for 5 min, and the final dissociation phase in running buffer was monitored for 10 min. Next, all bound analyte was removed with a 9 s pulse of 50 mM NaOH. Bulk differences between samples and running buffer due to DMSO were corrected using a DMSO titration curve. The assay was repeated for each 1.25, 2.5, 5, and 10 equiv of Ad–BSA. The binding profiles were double referenced (subtraction of FC1 signal and the average of triplicate buffer kinetic injections) to minimize the impact of instrument noise and baseline drift. Kinetic titration data (FC4) were fit globally to a Langmuir 1:1 binding model using Clamp (BioLogic Software) to calculate the apparent equilibrium dissociation constant (*K*_D_) and dissociation rate constants (*k*_d_); complex half-life was subsequently calculated according to eq 1.
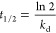
1

### Hydrogel Release Studies

Granular hydrogels (30 μL, *n* ≥ 4), loaded with fluorescently labeled BSA and Ad–BSA (0.5 mg/mL), were prepared and placed into the depressed region of custom-made acrylic erosion wells (4.3 mm diameter, 7 mm depth) below a buffer reservoir (1.6 cm diameter, 10 mm depth) to assess release. The wells were briefly centrifuged to level the hydrogel surface, and 1 mL of PBS was added to each well. The wells were incubated at 37 °C with buffer collected and replaced on days 1, 2, 4, and 7 and twice weekly thereafter. At end point, hydrogels were degraded in dextranase (50 U/mL) for complete recovery. BSA concentration was quantified relative to standard curves generated for the conjugates (Pacific Blue λ_ex/em_ = 410/455 nm; fluorescein λ_ex/em_ = 490/525 nm; AlexaFluor555 λ_ex/em_ = 555/580 nm) and reported as cumulative release, normalized to the total quantity recovered.

The release of modified (Ad-IL4, Ad-IL10, and Ad-IFNγ) or unmodified (IL-4, IL-10, and IFNγ) cytokines (15 μg IL-4 and IL-10; 40 μg IFNγ) from hydrogels (50 μL, *n* ≥ 3) was examined in 1 mL of media, incubated at 37 °C. Media were collected with replacement on days 1, 3, 7, 10, and 14. At the study end point, hydrogels were collected and pulverized using a pestle motor mixer (Thermo Fisher Scientific). All samples were stored at −80 °C until further analysis. Cytokine release was quantified by ELISA (mouse IL-10, IL-4, and IFNγ Quantikine kits, R&D Systems) according to manufacturer protocols. Data are presented as the total quantity released.

### Cytokine and Hydrogel Bioactivity

Ad-modified cytokines and their respective loaded hydrogels were assessed for bioactivity relative to unmodified control cytokines by the transcriptional analysis of treated bone marrow-derived macrophages (BMDMs). Animal procedures were performed in accordance with the guidelines for care and use of laboratory animals at Drexel University; procedures were approved by the University’s Institutional Animal Care and Use Committee (Protocol LA-22-056). To obtain BMDMs for transcriptional analysis, bone marrow was extracted according to established protocols.^52^ Briefly, the femur and tibia were isolated from C57BL/6 female mice; the marrow was collected by flushing with PBS and passed through a 70 μm strainer (Thermo Fisher Scientific). Red blood cells were lysed with ammonium-chloride-potassium (ACK) lysing buffer (Thermo Fisher Scientific), and resulting cells were plated at 1 × 10^6^ cells per mL in 24-well plates. Cells were differentiated under standard culture conditions (37 °C, 5% CO_2_) in Iscove’s Modified Dulbecco’s Medium (Cytiva) supplemented with 10% heat-inactivated fetal bovine serum (FBS), 1% penicillin/streptomycin, and 10 ng/mL recombinant murine macrophage colony-stimulating factor (M-CSF) for 7 days. Media were replenished every 2 days.

Resulting BMDMs were treated for 24 h with unmodified and modified cytokine (10 ng/mL IL-4, 10 ng/mL IL-10, or 50 ng/mL IFNγ), as well as the pulverized hydrogels (45 μL) with loaded cytokines suspended in 500 μL media. RNA was extracted (RNeasy kit; Qiagen), reverse transcribed (High-Capacity cDNA Reverse Transcription Kit; Thermo Fisher Scientific), and analyzed via qPCR (TaqMan Fast Advanced Master Mix; Thermo Fisher Scientific) for determining the levels of *Hprt* (Mm01545399_m1), *Mrc1* (Mm00485148_m1), *Arg1* (Mm00475988_m1), *Nos2* (Mm00440502_m1), and *Il1b* (Mm00434228_m1). Gene expression data are presented as fold change relative to *Hprt* and untreated (M0) controls by the ΔΔCt method.^53^

### Statistical Analysis

Data are presented as mean ± standard deviation (SD), unless otherwise indicated. Statistical analyses were performed with GraphPad Prism v9.5.0. Normality was confirmed by the Shapiro–Wilk test. Where appropriate, analysis of variance (ANOVA) was used with posthoc Tukey’s honest significant difference (HSD) test. For qPCR studies, a two-way ANOVA was performed and followed by Fisher’s least significant difference (LSD) posthoc test. For protein release data, temporal comparisons were made by repeated measures (RM) one-way or two-way ANOVA. Significance was determined at *P* < 0.05.

## Results and Discussion

### Chemical Modification of CD

**Figure 1:**
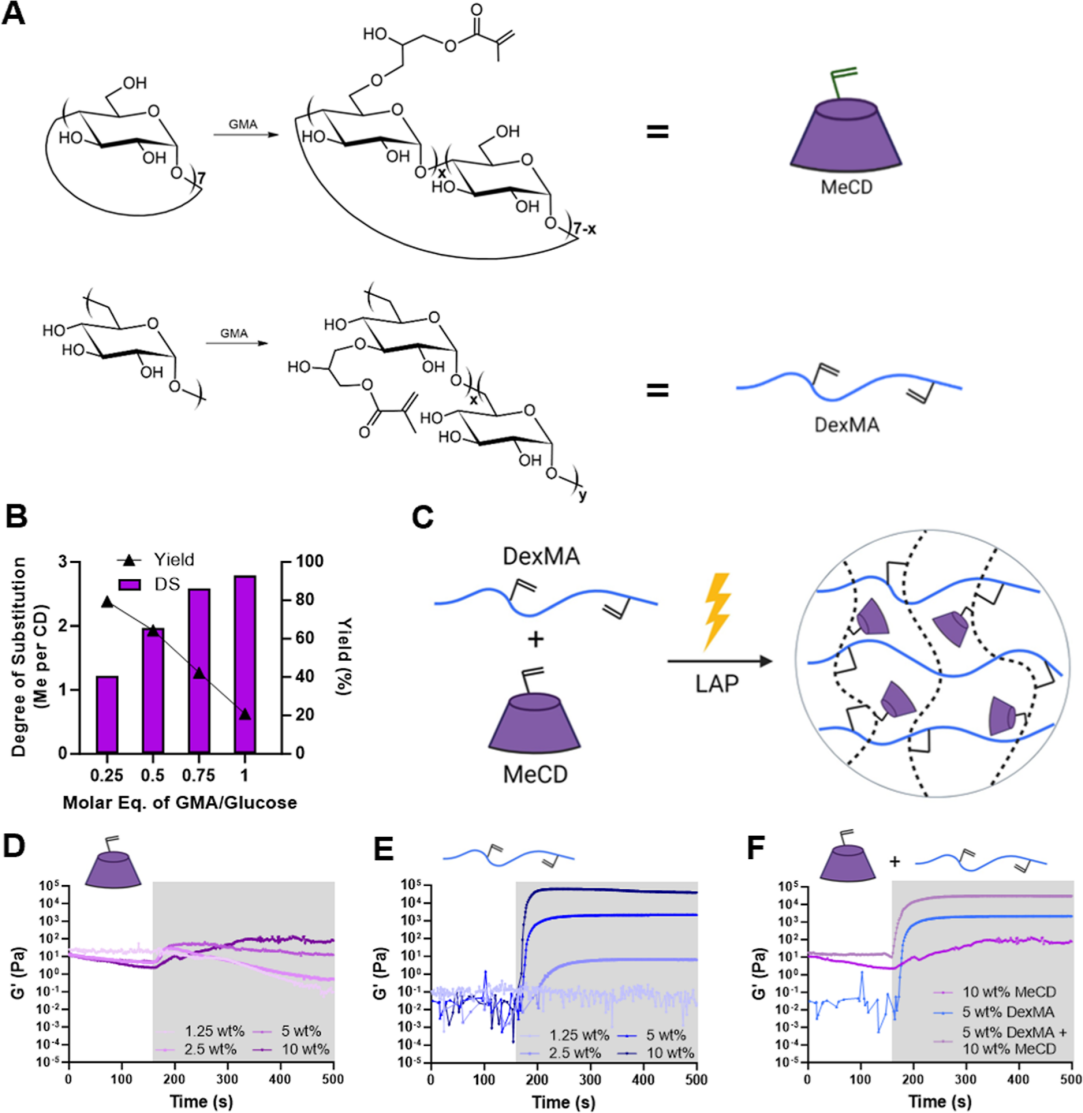
Synthesis and characterization of DexMA and MeCD. (A) Schematic of methacrylated cyclodextrin (MeCD, top, purple) and methacrylated dextran (DexMA, bottom, blue) synthesis through esterification with GMA. (B) DS and yield dependence on the molar feed ratio of GMA to glucose repeat units. (C) Hydrogels are formed through copolymerization of MeCD and DexMA. Representative oscillatory time sweeps (1 Hz, 1.0% strain) of photopolymerization (10 mW/cm^2^, 5 min as indicated by the shaded area) showing shear moduli (*G*′) of MeCD alone (D), DexMA alone (E), and their combination (F).

The pendant modification of polysaccharides, including by methacrylate groups, is a facile means of forming biopolymer-based hydrogels via photopolymerization. Specifically, the functionalization of polyglucose-based materials (e.g., dextran and CDs) has been achieved through base-driven esterification with methacrylic anhydride or GMA.^50,51,54^ Here, we used GMA modification under anhydrous conditions to synthesize a photocrosslinkable methacrylated-β-cyclodextrin (MeCD) host to allow for the formation of hydrogels with a high density of host binding sites (Figure 1A). The structure of MeCD was confirmed by ^1^H NMR spectroscopy (Figure S1A–D), and the DS was found to be roughly linearly dependent on the molar feed ratio of GMA to glucose (Figure 1B). A DS of 1.22, 1.97, 2.59, and 2.79 methacrylates per CD (corresponding to 0.25, 0.50, 0.75, and 1.00 molar feed ratios) were determined by integration of the methyl triplet (δ = 1.9, 3H) relative to the CD position 1 anomeric proton (δ = 4.8, 7H). However, increasing CD modification reduced reaction yields, which were, respectively, 79.5, 64.4, 42.3, and 20.8%. Products having a 1.97 DS were used for subsequent studies due to the combination of moderate yield and sufficient methacrylation for hydrogel inclusion. These results demonstrate controlled methacrylation of CD by adjusting the feed ratio of GMA, generating a base component for creating covalently cross-linked hydrogels composed of our host, in which CD is accessible for sequestration with our guest molecule.

### Bulk Hydrogel Formation

Storage (*G*′) and loss (*G*″) moduli were directly measured throughout photopolymerization (Figure 1C) by oscillatory shear rheology to confirm the formation of a bulk hydrogel. Despite methacrylation, MeCD alone failed to polymerize into a solid hydrogel with any appreciable storage modulus [*G*′ = 0.12 kPa, tan(δ) = 0.8 for 10%_w/v_ MeCD; tan(δ) > 1 for other MeCD concentrations; Figures 1D and S2A]. Notably, MeCD formed a white suspension after polymerization, indicative of aggregate formation in the absence of a continuous percolating network. This motivated the incorporation of DexMA as a copolymerization agent. DexMA (DS = 25%) was produced by an identical reaction with GMA. While DexMA lacks the necessary host functionality for sequestration of molecular guests exhibited by CD, it has a comparable polyglucose composition, necessary chain length for entanglement, and increased number of methacrylates per polymer chain, which are required for the formation of a solid percolating network. DexMA alone polymerized into a hydrogel at concentrations 2.5%_w/v_ or greater [tan(δ) ≪ 1; Figure S2B], with final moduli dependent on the polymer concentration (Figure 1E). Upon addition of 5%_w/v_ DexMA, copolymerization was observed for all MeCD concentrations (Figures 1F, S2C, and S3A,B), with final storage moduli (29.1 kPa) exceeding those of DexMA alone (2.2 kPa). Relative increases in *G*′ are attributable to chain entanglement of DexMA and the persistence length of kinetic chains formed in the presence of a higher methacrylate density. The use of MeCD in such a copolymerization scheme uniquely enables covalent inclusion of CD hosts within a continuous hydrogel network at high concentrations. For further studies, 5%_w/v_ DexMA and 10%_w/v_ MeCD were therefore used as the base material to allow for a high host capacity inside the hydrogels while enabling solid hydrogel formation.

### Granular Hydrogel Formation

**Figure 2:**
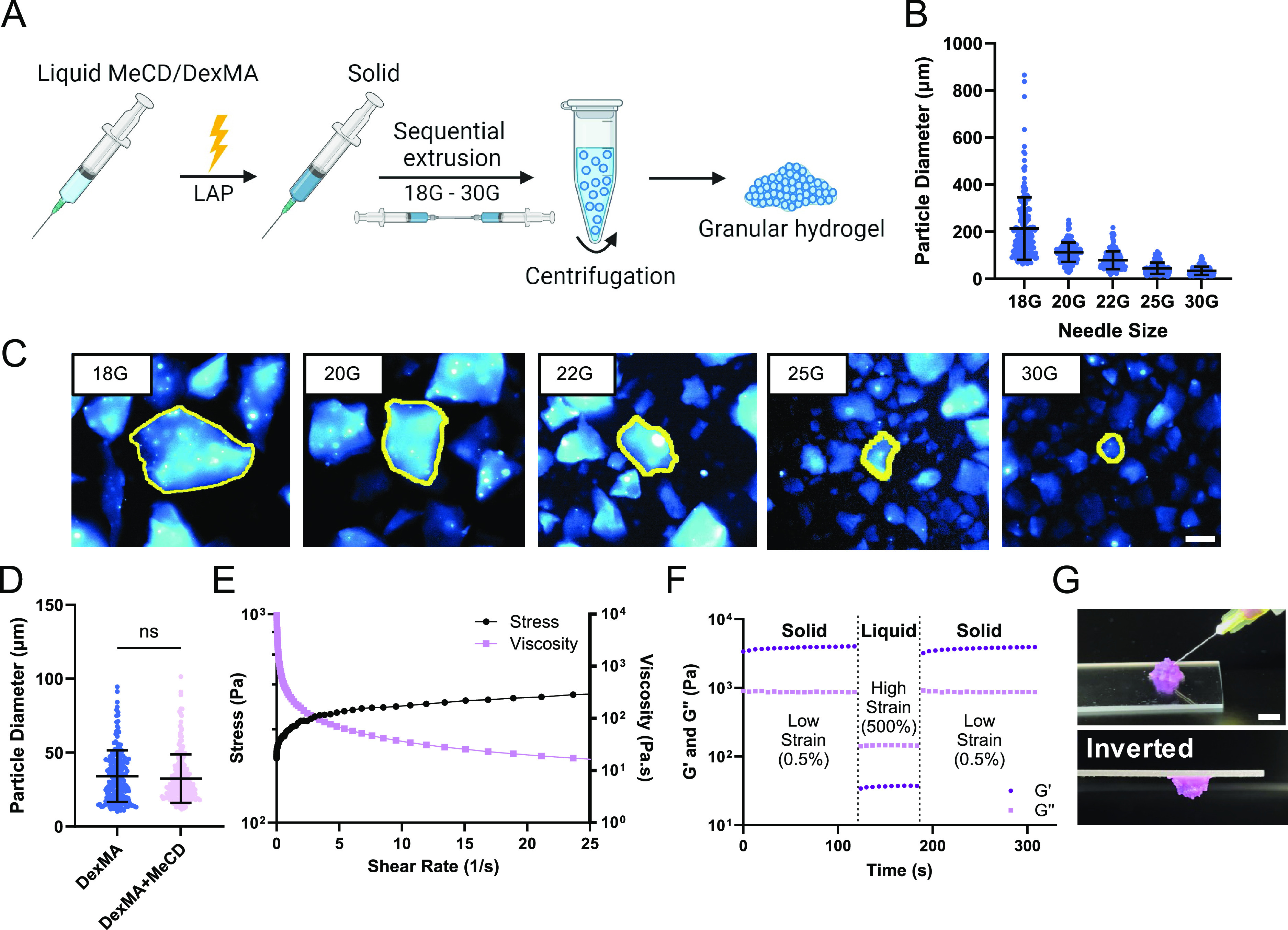
Granular hydrogel formation and characterization. (A) Schematic representation of microgel fabrication by EF. (B,C) Particle diameter throughout the extrusion process of 5%_w/v_ DexMA gels (mean ± SD; *n* = 200 particles), quantified using fluorescence microscopy images (C, scale bar = 100 μm). Representative particles are outlined (yellow) for clarity. (D) Final particle diameter of 5%_w/v_ DexMA and 5%_w/v_ DexMA +10%_w/v_ MeCD microgels (mean ± SD; *n* = 200; ns = not significant). (E) Continuous flow experiments showing the shear stress and viscosity of 5%_w/v_ DexMA +10%_w/v_ MeCD granular hydrogels. (F) Cyclic deformation at low (0.5%) and high (500%) strain (1.0 Hz) of 5%_w/v_ DexMA + 10%_w/v_ MeCD hydrogels; *G*′ (storage modulus, dark purple, circle), *G*″ (loss modulus, light purple, circle). (G) Representative images of granular hydrogel injection (30G needle, 1 mL syringe; scale bar = 5 mm).

Bulk hydrogels were subsequently processed into granular hydrogels to allow for injectable delivery (Figure 2A). For ease of scalable production while avoiding residual oil or surfactants commonly used in bulk emulsification, we elected to generate microgels by EF, similar to reported protocols.^36^ Hydrogels (5%_w/v_ DexMA) were cast directly in the syringe and sequentially extruded through progressively narrowing emulsification needles (18–30G), requiring minimal mechanical force. Granules after 18G extrusion were large, as imaging of fluorescently tagged particles exhibited a wide size distribution (215.4 ± 109.7 μm) with particles becoming progressively smaller and more homogeneous throughout the extrusion process (Figure 2B,C). Qualitatively, composite (DexMA + MeCD) hydrogels required greater mechanical force for extrusion compared to DexMA-alone hydrogels, but they fragmented into similarly sized microgels (DexMA: 34.1 ± 17.5 μm; DexMA + MeCD: 32.4 ± 16.4 μm diameter; Figures 2D and S4A). Final composite microgels were irregularly shaped, with a circularity of 0.2 ± 0.1 (Figure S4B) and an aspect ratio of 1.6 ± 0.5 (Figure S4C). Importantly, these composite granular hydrogels contain the host evenly dispersed throughout, providing a high host capacity for included therapeutics.

The moduli of the granular hydrogels (*G*′ and *G*″) were both substantially reduced, compared to those of their bulk counterparts (Figure S4D). However, granular hydrogels composed of DexMA alone or including MeCD were solids as a result of interparticle jamming.^35,55^ Granular media, including hydrogels, frequently exhibit deformation and shear-thinning flow under high strain conditions (such as flow through a needle) but rapidly regain their mechanical integrity after force is removed (such as after injection in tissue) as a result of particle jamming.^39,56^ Under ramped flow conditions, the hydrogels exhibited decreased viscosity and a corresponding plateau in shear stress, characteristic of a shear-thinning behavior (Figure 2E). Moduli were further examined under alternating low and high strain conditions. Granular hydrogels quickly transitioned from a solid (*G*′ > *G*″) to fluid-like (*G*″ > *G*′) state at high strain, subsequently recovering their storage moduli (>95% recovery in 7 s) rapidly upon the removal of high strain (Figure 2F). This strain-yielding behavior was consistent with oscillatory strain sweeps, which showed a yield point of 44% (Figure S4E). These findings demonstrated a shear-thinning and self-healing hydrogel suited for local delivery via a minimally invasive injection (Figure 2G). While covalently cross-linked hydrogels themselves are not injectable and would require invasive implantation, processing into granular hydrogels enables injectable delivery that can be used to localize the materials and their cargo easily within a tissue of interest.

### Protein Modification

**Figure 3:**
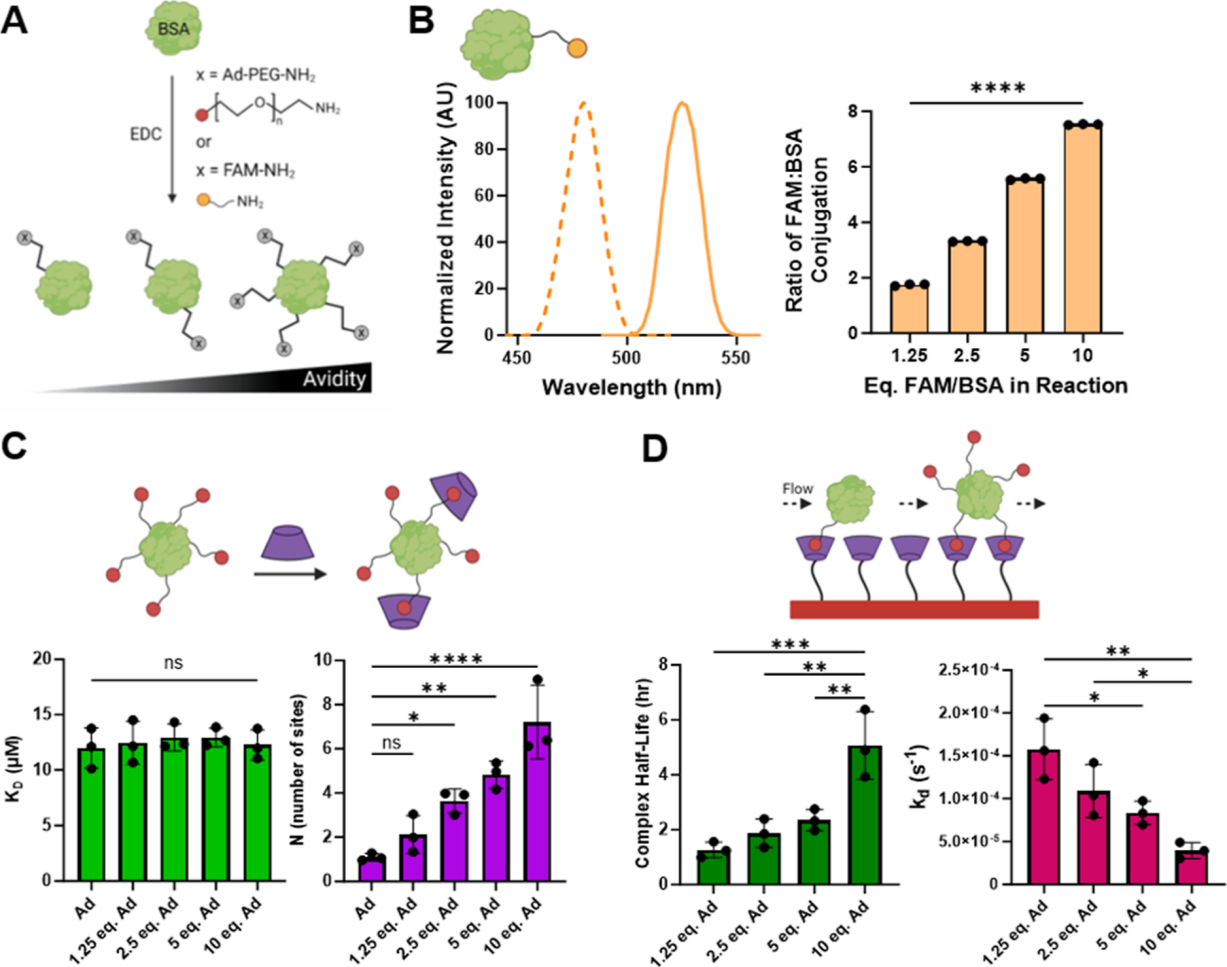
Chemical modification of BSA. (A) Schematic of BSA modification with FAM and/or Ad–PEG–amine via EDC-catalyzed amidation. (B) FAM–BSA excitation and emission scans (λ_max ex/em_ = 480/525 nm, left). Dependence of the FAM-per-BSA modification ratio on the molar feed ratio of FAM to BSA (right). Differences between all reaction conditions were highly significant, ****P < 0.0001. (C) The affinity-based thermodynamic dissociation constant of individual GH interactions (*K*_D_, bottom left) and extent of Ad–BSA modification (N, bottom right), determined by ITC (see Figures S5 and S6). (D) Avidity-controlled Ad–BSA complex half-life (bottom left) and dissociation rate constant (bottom right), determined by SPR. Data represent mean ± SD; *n* = 3; ns = not significant, *P < 0.05, **P < 0.01, ***P < 0.001, ****P < 0.0001; ANOVA, Tukey HSD.

The sustained local delivery of therapeutics is often desirable^57^ and may be accomplished through the use of specific supramolecular interactions between a material scaffold and small molecule^30,58,59^ or biomolecular^31,60,61^ cargo. Here, we specifically leverage the GH interaction between Ad and CD, where avidity of the guest-modified protein (controlled by the number of conjugated guests) controls release. For an initial proof-of-concept demonstration of the strategy, BSA, a model biomolecule, was modified using EDC-catalyzed amidation (Figure 3A).

To quantitatively assess BSA conjugation efficiency, we first used FAM as a stand-in for the Ad conjugate. Conjugation did not affect FAM fluorescence spectra that were used to quantify BSA coupling, and the extent of BSA conjugation was directly dependent on the FAM/BSA molar feed ratio (Figure 3B). Near-quantitative coupling was observed. Having confirmed that amidation enabled tunable control of protein modification, Ad was similarly conjugated to BSA with the inclusion of a 5 kDa amine-terminated PEG linker to maximize solubility and minimize steric hindrance that may otherwise obstruct desired GH interaction between Ad-functionalized proteins and host CD macrocycles. Ad conjugation was confirmed by ITC, where the binding stoichiometry (*n*) between CD and Ad increased in proportion to the feed ratio, ranging from 1.10 ± 0.17 to 7.22 ± 1.67 sites per BSA (Figure 3C).

Prior reports have indicated that guest modification of polymers or proteins can detrimentally affect GH affinity.^62,63^ Therefore, it was critical to determine whether the affinity between Ad and CD is hindered by Ad–protein conjugation. The affinity between Ad–BSA conjugates and CD was assessed by ITC. Unmodified BSA exhibited no interaction with CD, as seen in previous reports (Figure S5A).^64^ We discovered that a tighter fit of the isotherm depended on Ad concentration, as 10 equiv of Ad–BSA resulted in the greatest heat generation. An increase in enthalpy (Δ*H*) and entropy (Δ*S*) was furthermore observed (Figures S5B and S6A–D), consistent with an increased number of binding interactions between CD and Ad. The interaction affinity, however, was independent of BSA conjugation (Figure 3C), with thermodynamic dissociation constants (*K*_D_) ranging from 12.00 ± 1.81 to 12.94 ± 1.21 μM. These results demonstrate controlled modification of BSA through EDC chemistry, which does not impact affinity, making Ad–BSA suitable for delivery from CD.

The summation of multiple physical interactions can result in cooperative avidity that exceeds the strength of a single GH bond. The equilibrium dissociation constant (*K*_D_) controls protein release and is avidity dependent, being influenced by the number of simultaneous interactions possible.^65^ Ad–BSA avidity was therefore measured by SPR, which demonstrated a greater than twofold decrease in *K*_D_ from 356 to 164 nM with increasing guest-modification of BSA, reflecting a higher avidity with more Ad conjugation (Table S1). This trend is similar to previous reports, where Ad tethers resulted in an increase in affinity groups.^30,31^ The complex half-life also increased substantially by fourfold (from 1.27 ± 0.29 to 5.07 ± 1.23 h) with increasing Ad content—indicating that Ad–BSA is more rapidly cleared when less Ad is present (Figure 3D). While GH complexes can rapidly form, dissociate, and reform to allow for the clearance of guest species with low valence, higher guest valency results in the formation of multiple interactions that act cooperatively through avidity. This increases their interaction time and statistical likelihood of rebinding with host-substrates but does not change the individual bonds themselves. This avidity is important for sustained release, as control of clearance over time is essential for in vivo persistence and long-term therapeutic presentation.

In the presence of CD, Ad is driven toward complex formation, meaning that the high affinity ensures specific binding and relatively slow dissociation. Both the maintenance of the pair’s native affinity and the capacity to control avidity through molecular valence support the notion that our Ad-modified proteins would be suitable for inclusion inside of a host-modified hydrogel to achieve tunable release.

### Protein Release

**Figure 4:**
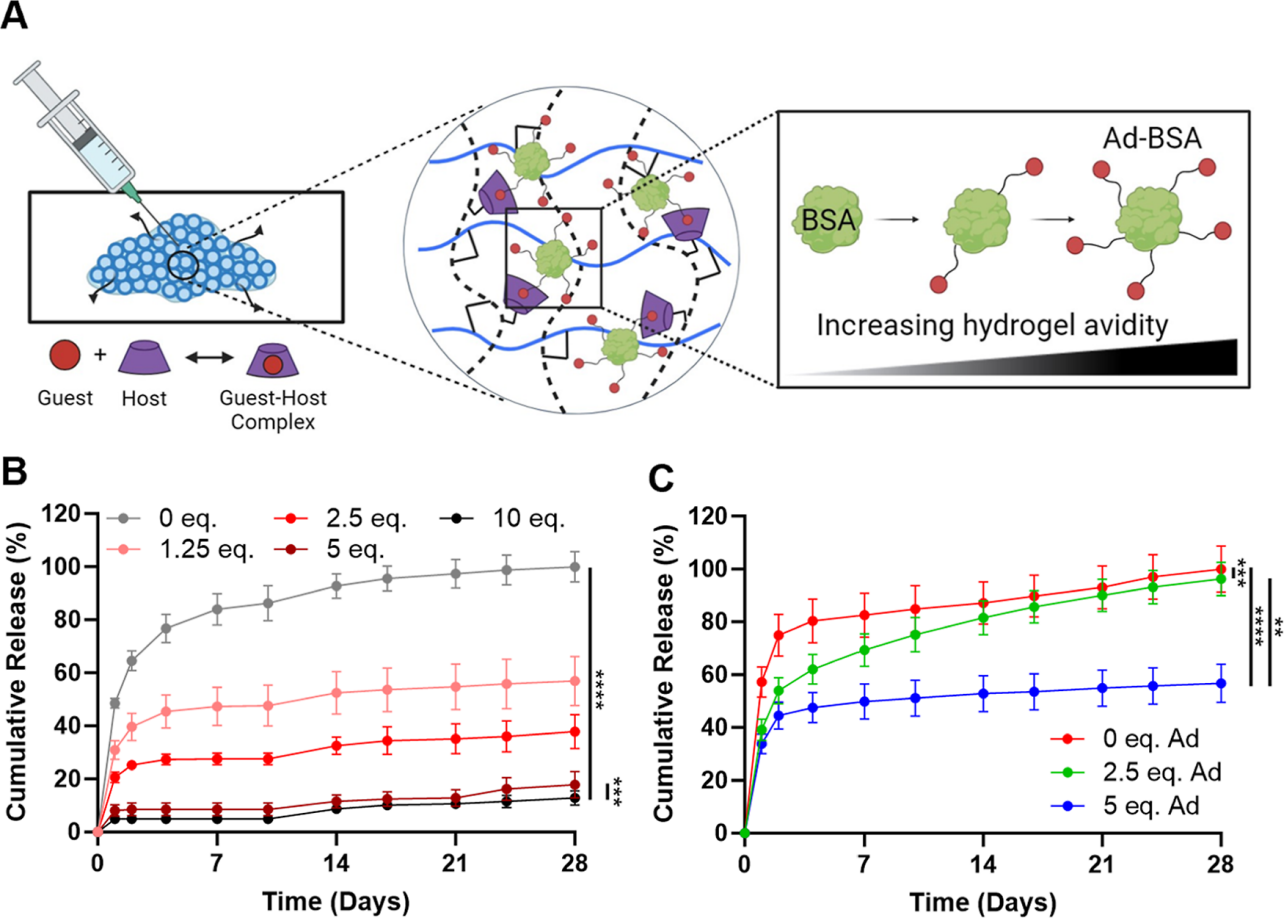
Model biomolecule release. (A) Schematic of biomolecule retention within the granular host hydrogels as a result of avidity-based interaction. (B) Cumulative release of Ad-FAM-BSA (0–10 equiv Ad); *n* = 4. (C) Controlled release of multiple components from the same hydrogel, including BSA-AlexaFluor555 (0 equiv Ad), Ad–BSA–fluorescein (2.5 equiv Ad), and Ad–BSA-Pacific Blue (5 equiv Ad); *n* = 6. Data represent the mean ± SD; **P < 0.01, ***P < 0.001, ****P < 0.0001; RM one-way ANOVA, Tukey HSD.

To demonstrate the avidity-controlled release of guest-modified protein cargo, they were included in granular host hydrogels (Figure 4A). Release kinetics for unmodified fluorescently labeled BSA (FAM–BSA) and BSA with increasing guest modifications (Ad–FAM–BSA) were monitored in vitro for 4 weeks. FAM–BSA exhibited a burst release profile, with complete release by the study end point. At high Ad-conjugate densities (5, 10 equiv), we observed a greater than fivefold reduction in the cumulative release of Ad–FAM–BSA, relative to FAM–BSA alone (Figure 4B). The extent of BSA release was directly dependent on the extent of guest modification, reflecting the GH interactions inside the hydrogel. BSA with 5 and 10 equiv of Ad behaved similarly but resulted in a significant difference, in accordance with their substantial differences in complex half-life observed in 2D (SPR, Figure 3D). This finding may be attributable to complex differences in binding within a 3D environment, wherein the whole of the protein surface is able to interact with the host component. Importantly, this change in biomolecule avidity increased the effective bonding interaction, independent of individual GH interaction affinity, and allowed for tunable retention and sustained release of the included biomolecule.

We further investigated the potential control of the release of multiple factors from a single hydrogel alone via several different fluorescently labeled BSA molecules. We discovered that an increase in the level of Ad conjugation was followed by an attenuation in the level of burst release. For unmodified BSA, 80% of the protein was released by day 4 (Figure 4C). In contrast, for BSA conjugated to 2.5 equiv of Ad, steady release (3–8%) was observed after day 2; BSA conjugated to 5 equiv of Ad followed a similar pattern (1–3% release) (Figure 4C). These results demonstrated controlled release of multiple components from a single hydrogel construct. We hypothesize that competitive binding underlies the change in release pattern, where BSA with multiple linkages outcompetes its rivals, consequently being sequestered for a longer duration. Therefore, release can be fine-tuned based on Ad conjugation, as the inherent GH interactions within the hydrogel provide a supramolecular network capable of modulating avidity and hence the delivery of model biomolecules.

### Cytokine Functionality and Release

**Figure 5:**
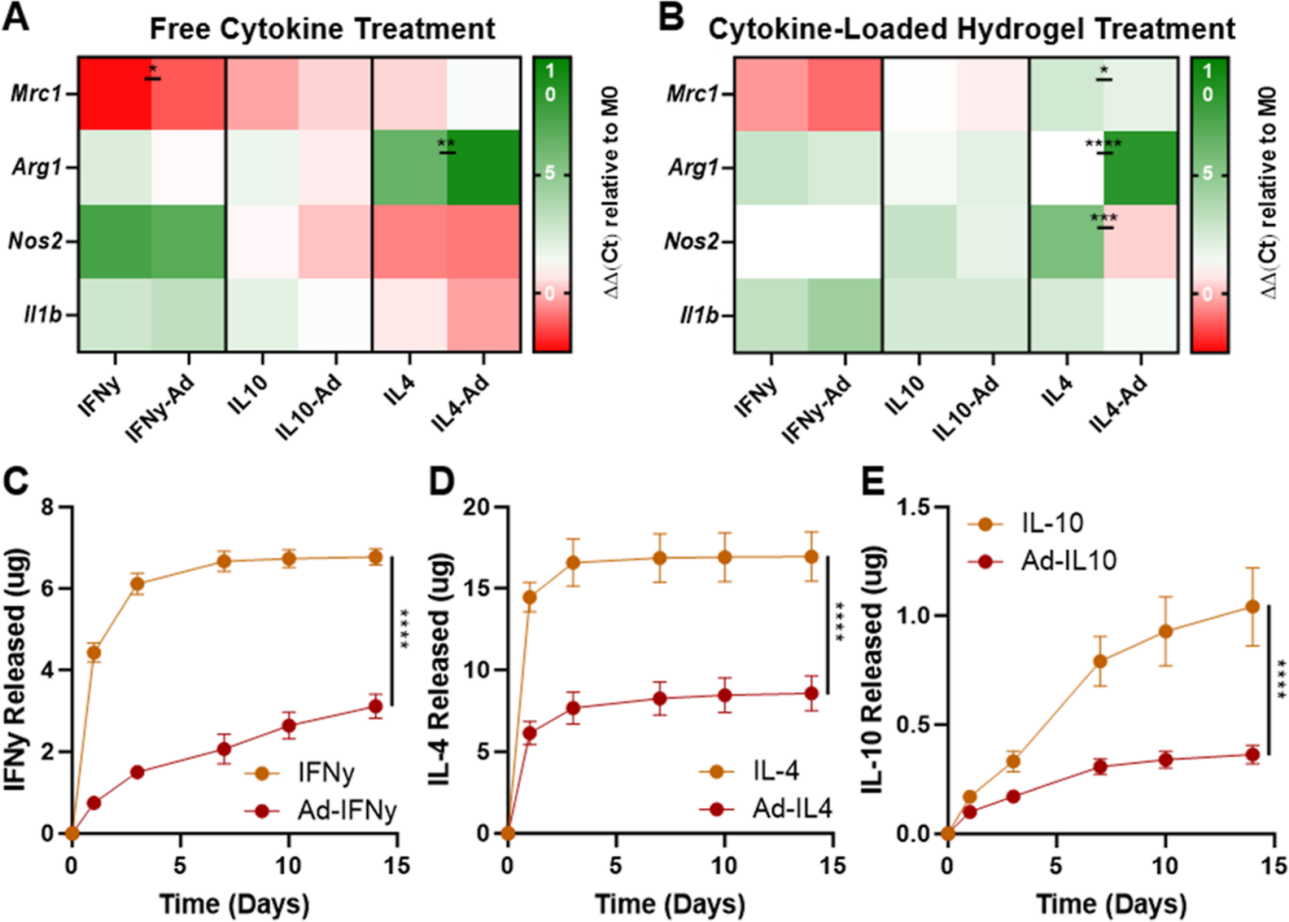
Cytokine functionality and release profile. Heat maps of gene expression levels in bone marrow-derived macrophages (BMDMs) after treatment with modified and unmodified cytokines (A) or final hydrogels at the end of 14 day release (B). Data represent mean ± SD; *n* ≥ 1; ns = not significant, *P < 0.05, **P < 0.01, ***P < 0.001, ****P < 0.0001; two-way ANOVA, Fisher LSD. Cytokine release profiles of unmodified and Ad-modified (5 equiv) IL-10 (C), IFNγ (D), and IL-4 (E) from DexMA + MeCD hydrogels into media. Data represent the mean ± SD; *n* ≥ 3; ****P < 0.0001; RM two-way ANOVA.

The chemical modification of biotherapeutics, and proteins in particular, is often complicated by a loss of biomolecule functionality subsequent to modification.^66^ We therefore examined the effect of our bioconjugation strategy on the activity of cytokines, which are moderately sized cell-signaling proteins often leveraged in immune modulation to treat cancer, autoimmune disorders, and infectious diseases.^67^ To confirm that our guest modification does not affect bioactivity, we modified a selection of frequently used cytokines (IL-10, IL-4, and IFNγ) with Ad and observed their effect on macrophage phenotype by treating BMDMs with unmodified and guest-modified cytokines (Figure 5A), including after prolonged inclusion within the microgels (Figure 5B). IFNγ is a potent proinflammatory cytokine that drives macrophages toward an M1-like (inflammatory) polarization, reflected by upregulation of canonical markers (e.g., *Nos2* and *Il1b*).^68−70^ On the other hand, IL-4 is known to suppress inflammation, polarizing macrophages toward an M2-like (anti-inflammatory) phenotype that is associated with *Mrc1* and *Arg1* expression.^71−74^ When treated with IFNγ or Ad-IFNγ, macrophages were driven toward an M1-like phenotype, including downregulation of *Mrc1* and an upregulation of *Nos2* and *Il1b* (Figure 5A). In contrast, when macrophages were treated with IL-4 or Ad-IL4, they were driven toward an M2-like phenotype, denoted by the upregulation of *Arg1* and moderate downregulation of *Nos2*. IL-10 is known for its pleiotropic functionality, as it possesses both inflammatory and anti-inflammatory properties. IL-10 can mediate inflammation by suppressing pro-inflammatory cytokine production and macrophage antigen presentation but, in response to immunosuppression, can upregulate IFNγ production.^75−78^ When treated with IL-10 or Ad-IL10, we observed a moderate upregulation of *Il1b*, consistent with previous reports (Figure 5A).^22,79^ Overall, the guest-modified cytokines behaved in a comparable manner to unmodified cytokines, indicating that modification does not affect their bioactivity either as a result of prohibiting receptor interaction or via indirect effects of the PEG-Ad conjugate.

Having observed the retained bioactivity of Ad-modified cytokines, we next sought to examine their controlled release (Figure 5C–E). Unmodified control cytokines typically exhibited a rapid burst release with 90 and 98% release observed by day 3 for IFNγ and IL-4, respectively. In comparison, the IL-10 release was moderately delayed. In all cases, guest modification resulted in a more controlled release, with a greater than twofold reduction in cumulative release (relative to controls) by day 14. Differences in the release kinetics between each of these cytokines and BSA may partly be attributed to their relative size as cytokines are notably smaller than BSA (66 kDa), with IL-4 (13.5 kDa) being the smallest relative to IFNγ (15.6 kDa) and IL-10 (18.6 kDa). It may be expected that the relatively small hydrodynamic radius of cytokines affects not only their inherent diffusivity within the hydrogel but also their capacity to form multivalent bonds within the host-hydrogel network. Inclusion of the PEG linker likely contributes to the ability to overcome these considerations of spatial restriction to allow for GH binding. Nonetheless, guest modification of the cytokines reduced burst release and retained the cytokine inside the host hydrogel for a prolonged period of time.

Throughout this period, the hydrogel-bound cytokines retained their bioactivity. At the end point of cytokine release studies, macrophages treated with the collected cytokine-loaded microgels responded similarly to treatment by the parental cytokines examined above. Of note, cells treated with Ad-IFNγ hydrogels exhibited a more pronounced downregulation of *Mrc1* and upregulation of *Il1b* compared to the control hydrogels. Additionally, in the case of the Ad-IL10 and Ad-IL4 hydrogels, macrophages were continually driven toward an M2-like state, as there was a decrease in *Nos2* and *Il1b* expression relative to the controls (Figure 5B). These results indicate that Ad-modified cytokines remained bound to CD within the hydrogels and maintained their bioactivity for greater than 2 weeks. These results are promising, as they illustrate the potential of our hydrogel system to control the localized presentation of bioactive cytokines for prolonged periods of time, which can bolster the timespan of therapeutic response while limiting off-target effects.

## Conclusions

In conclusion, we have demonstrated the potential of a unique affinity-based hydrogel delivery system that leverages supramolecular interactions to enable sustained release of biotherapeutics. Chemical modification of biomolecules and cytokines via EDC chemistry enabled a controlled method for conjugating a guest moiety (Ad) to proteins. A granular hydrogel system composed of DexMA and MeCD was utilized for controllably releasing Ad-modified proteins, taking advantage of the Ad–CD complexation. By incorporation of a high-avidity host network inside the hydrogels, protein release was readily tunable according to the extent of Ad conjugation, which directly altered the supramolecular avidity of proteins to the host network. Granular hydrogels formed by EF are shear-thinning, which allows them to be locally injected in vivo for therapeutic applications. Importantly, the hydrogel system enables controlled and sustained release of biomolecules that can be applied to modulate tissue response in various pathological contexts such as cancer and autoimmune diseases.

## Supporting Information Available

^1^H NMR spectra of MeCD, real-time rheological assessment of photopolymerization at varying component concentrations, additional characterization of granular hydrogel properties and dependence on composition, ITC thermograms for negative (BSA) and positive (Ad) binding to CD, and representative ITC thermograms for Ad–BSA conjugates (PDF)

## References

[ref1] Usmani S. S.; Bedi G.; Samuel J. S.; Singh S.; Kalra S.; Kumar P.; Ahuja A. A.; Sharma M.; Gautam A.; Raghava G. P. S. THPdb: Database of FDA-approved peptide and protein therapeutics. PLoS One 2017, 12 (7), e0181748 10.1371/journal.pone.0181748. 28759605 PMC5536290

[ref2] Leader B.; Baca Q. J.; Golan D. E. Protein therapeutics: a summary and pharmacological classification. Nat. Rev. Drug Discovery 2008, 7 (1), 21–39. 10.1038/nrd2399. 18097458

[ref3] Lagassé H. D.; Alexaki A.; Simhadri V. L.; Katagiri N. H.; Jankowski W.; Sauna Z. E.; Kimchi-Sarfaty C. Recent advances in (therapeutic protein) drug development. F1000Research 2017, 6, 113 10.12688/f1000research.9970.1. 28232867 PMC5302153

[ref4] Correa S.; Grosskopf A. K.; Klich J. H.; Lopez Hernandez H.; Appel E. A. Injectable Liposome-based Supramolecular Hydrogels for the Programmable Release of Multiple Protein Drugs. Matter 2022, 5 (6), 1816–1838. 10.1016/j.matt.2022.03.001. 35800848 PMC9255852

[ref5] Foster G. A.; Headen D. M.; González-García C.; Salmerón-Sánchez M.; Shirwan H.; García A. J. Protease-degradable microgels for protein delivery for vascularization. Biomaterials 2017, 113, 170–175. 10.1016/j.biomaterials.2016.10.044. 27816000 PMC5121008

[ref6] Grosso A.; Lunger A.; Burger M. G.; Briquez P. S.; Mai F.; Hubbell J. A.; Schaefer D. J.; Banfi A.; Di Maggio N. VEGF dose controls the coupling of angiogenesis and osteogenesis in engineered bone. npj Regener. Med. 2023, 8 (1), 15 10.1038/s41536-023-00288-1. PMC10011536 36914692

[ref7] Purcell B. P.; Barlow S. C.; Perreault P. E.; Freeburg L.; Doviak H.; Jacobs J.; Hoenes A.; Zellars K. N.; Khakoo A. Y.; Lee T.; Burdick J. A.; Spinale F. G. Delivery of a matrix metalloproteinase-responsive hydrogel releasing TIMP-3 after myocardial infarction: effects on left ventricular remodeling. Am. J. Physiol. Heart Circ. Physiol. 2018, 315 (4), H814–h825. 10.1152/ajpheart.00076.2018. 29979624 PMC6230910

[ref8] Partain B. D.; Bracho-Sanchez E.; Farhadi S. A.; Yarmola E. G.; Keselowsky B. G.; Hudalla G. A.; Allen K. D. Intra-articular delivery of an indoleamine 2,3-dioxygenase galectin-3 fusion protein for osteoarthritis treatment in male Lewis rats. Arthritis Res. Ther. 2023, 25 (1), 173 10.1186/s13075-023-03153-0. 37723593 PMC10506271

[ref9] An Y.-H.; Park M. J.; Lee J.; Ko J.; Kim S.-H.; Kang D. H.; Hwang N. S. Recent Advances in the Transdermal Delivery of Protein Therapeutics with a Combinatorial System of Chemical Adjuvants and Physical Penetration Enhancements. Adv. Ther. 2020, 3 (2), 1900116 10.1002/adtp.201900116.

[ref10] Rosenberg A. S.; Sauna Z. E. Immunogenicity assessment during the development of protein therapeutics. J. Pharm. Pharmacol. 2018, 70 (5), 584–594. 10.1111/jphp.12810. 28872677

[ref11] Yin L.; Yuvienco C.; Montclare J. K. Protein based therapeutic delivery agents: Contemporary developments and challenges. Biomaterials 2017, 134, 91–116. 10.1016/j.biomaterials.2017.04.036. 28458031 PMC5513498

[ref12] Rizzo F.; Kehr N. S. Recent Advances in Injectable Hydrogels for Controlled and Local Drug Delivery. Adv. Healthcare Mater. 2021, 10 (1), e2001341 10.1002/adhm.202001341. 33073515

[ref13] Chen Y.-C.; Gad S. F.; Chobisa D.; Li Y.; Yeo Y. Local drug delivery systems for inflammatory diseases: Status quo, challenges, and opportunities. J. Controlled Release 2021, 330, 438–460. 10.1016/j.jconrel.2020.12.025. 33352244

[ref14] Vulic K.; Shoichet M. S. Affinity-Based Drug Delivery Systems for Tissue Repair and Regeneration. Biomacromolecules 2014, 15 (11), 3867–3880. 10.1021/bm501084u. 25230248

[ref15] Varshey D. B.; Sander J. R. G.; Friščić T.; MacGillivray L. R. Supramolecular chemistry: from molecules to nanomaterials. Supramolecular Interactions; John Wiley & Sons, Ltd: Chichester, UK, 2012.

[ref16] Webber M. J.; Langer R. Drug delivery by supramolecular design. Chem. Soc. Rev. 2017, 46 (21), 6600–6620. 10.1039/c7cs00391a. 28828455

[ref17] Park J.; Park J.; Lee J.; Lim C.; Lee D. W. Size compatibility and concentration dependent supramolecular host-guest interactions at interfaces. Nat. Commun. 2022, 13 (1), 112 10.1038/s41467-021-27659-w. 35013244 PMC8748952

[ref18] Mukhopadhyay R. D.; Das G.; Ajayaghosh A. Stepwise control of host-guest interaction using a coordination polymer gel. Nat. Commun. 2018, 9 (1), 1987 10.1038/s41467-018-04303-8. 29777098 PMC5959896

[ref19] Zou L.; Braegelman A. S.; Webber M. J. Dynamic Supramolecular Hydrogels Spanning an Unprecedented Range of Host-Guest Affinity. ACS Appl. Mater. Interfaces 2019, 11 (6), 5695–5700. 10.1021/acsami.8b22151. 30707553

[ref20] Mealy J. E.; Rodell C. B.; Burdick J. A. Sustained Small Molecule Delivery from Injectable Hyaluronic Acid Hydrogels through Host-Guest Mediated Retention. J. Mater. Chem. B 2015, 3 (40), 8010–8019. 10.1039/C5TB00981B. 26693019 PMC4675358

[ref21] Rodell C. B.; Kaminski A. L.; Burdick J. A. Rational Design of Network Properties in Guest-Host Assembled and Shear-Thinning Hyaluronic Acid Hydrogels. Biomacromolecules 2013, 14 (11), 4125–4134. 10.1021/bm401280z. 24070551 PMC3851010

[ref22] Rodell C. B.; Arlauckas S. P.; Cuccarese M. F.; Garris C. S.; Li R.; Ahmed M. S.; Kohler R. H.; Pittet M. J.; Weissleder R. TLR7/8-agonist-loaded nanoparticles promote the polarization of tumour-associated macrophages to enhance cancer immunotherapy. Nat. Biomed. Eng. 2018, 2 (8), 578–588. 10.1038/s41551-018-0236-8. 31015631 PMC6192054

[ref23] Soni S. S.; D’Elia A. M.; Alsasa A.; Cho S.; Tylek T.; O’Brien E. M.; Whitaker R.; Spiller K. L.; Rodell C. B. Sustained release of drug-loaded nanoparticles from injectable hydrogels enables long-term control of macrophage phenotype. Biomater. Sci. 2022, 10 (24), 6951–6967. 10.1039/d2bm01113a. 36341688 PMC9724601

[ref24] Kakuta T.; Takashima Y.; Nakahata M.; Otsubo M.; Yamaguchi H.; Harada A. Preorganized Hydrogel: Self-Healing Properties of Supramolecular Hydrogels Formed by Polymerization of Host-Guest-Monomers that Contain Cyclodextrins and Hydrophobic Guest Groups. Adv. Mater. 2013, 25 (20), 2849–2853. 10.1002/adma.201205321. 23423947

[ref25] Rodell C. B.; Mealy J. E.; Burdick J. A. Supramolecular Guest-Host Interactions for the Preparation of Biomedical Materials. Bioconjugate Chem. 2015, 26 (12), 2279–2289. 10.1021/acs.bioconjchem.5b00483. 26439898

[ref26] Alvarez-Lorenzo C.; García-González C. A.; Concheiro A. Cyclodextrins as versatile building blocks for regenerative medicine. J. Controlled Release 2017, 268, 269–281. 10.1016/j.jconrel.2017.10.038. 29107127

[ref27] Wankar J.; Kotla N. G.; Gera S.; Rasala S.; Pandit A.; Rochev Y. A. Recent Advances in Host-Guest Self-Assembled Cyclodextrin Carriers: Implications for Responsive Drug Delivery and Biomedical Engineering. Adv. Funct. Mater. 2020, 30 (44), 1909049 10.1002/adfm.201909049.

[ref28] Yi S.; Liao R.; Zhao W.; Huang Y.; He Y. Multifunctional co-transport carriers based on cyclodextrin assembly for cancer synergistic therapy. Theranostics 2022, 12 (6), 2560–2579. 10.7150/thno.70243. 35401819 PMC8965498

[ref29] Granadero D.; Bordello J.; Pérez-Alvite M. J.; Novo M.; Al-Soufi W. Host-guest complexation studied by fluorescence correlation spectroscopy: adamantane-cyclodextrin inclusion. Int. J. Mol. Sci. 2010, 11 (1), 173–188. 10.3390/ijms11010173. 20162009 PMC2820997

[ref30] Dogan A.; von Recum H. Engineering selective molecular tethers to enhance suboptimal drug properties. Acta Biomater. 2020, 115, 383–392. 10.1016/j.actbio.2020.07.045. 32846237

[ref31] Dogan A. B.; Dabkowski K. E.; von Recum H. A. Leveraging Affinity Interactions to Prolong Drug Delivery of Protein Therapeutics. Pharmaceutics 2022, 14 (5), 1088 10.3390/pharmaceutics14051088. 35631672 PMC9144912

[ref32] Addonizio C. J.; Gates B. D.; Webber M. J. Supramolecular “Click Chemistry” for Targeting in the Body. Bioconjugate Chem. 2021, 32 (9), 1935–1946. 10.1021/acs.bioconjchem.1c00326. PMC8764705 34415139

[ref33] Ooi H. W.; Kocken J. M. M.; Morgan F. L. C.; Malheiro A.; Zoetebier B.; Karperien M.; Wieringa P. A.; Dijkstra P. J.; Moroni L.; Baker M. B. Multivalency Enables Dynamic Supramolecular Host-Guest Hydrogel Formation. Biomacromolecules 2020, 21 (6), 2208–2217. 10.1021/acs.biomac.0c00148. 32243138 PMC7284802

[ref34] Qazi T. H.; Burdick J. A. Granular hydrogels for endogenous tissue repair. Biomater. Biosyst. 2021, 1, 100008 10.1016/j.bbiosy.2021.100008. 36825161 PMC9934473

[ref35] Riley L.; Schirmer L.; Segura T. Granular hydrogels: emergent properties of jammed hydrogel microparticles and their applications in tissue repair and regeneration. Curr. Opin. Biotechnol. 2019, 60, 1–8. 10.1016/j.copbio.2018.11.001. 30481603 PMC6534490

[ref36] Muir V. G.; Qazi T. H.; Shan J.; Groll J.; Burdick J. A. Influence of Microgel Fabrication Technique on Granular Hydrogel Properties. ACS Biomater. Sci. Eng. 2021, 7 (9), 4269–4281. 10.1021/acsbiomaterials.0c01612. 33591726 PMC8966052

[ref37] Hirsch M.; Charlet A.; Amstad E. 3D Printing of Strong and Tough Double Network Granular Hydrogels. Adv. Funct. Mater. 2021, 31 (5), 2005929 10.1002/adfm.202005929.

[ref38] Wang W.; Chen X.; Meng T.; Liu L. Multi-network granular hydrogel with enhanced strength for 3D bioprinting. J. Biomater. Appl. 2022, 36 (10), 1852–1862. 10.1177/08853282221075198. 35225041

[ref39] Emiroglu D. B.; Bekcic A.; Dranseike D.; Zhang X.; Zambelli T.; deMello A. J.; Tibbitt M. W. Building block properties govern granular hydrogel mechanics through contact deformations. Sci. Adv. 2022, 8 (50), eadd8570 10.1126/sciadv.add8570. 36525484 PMC9757745

[ref40] Liu Y.; Suarez-Arnedo A.; Riley L.; Miley T.; Xia J.; Segura T. Spatial Confinement Modulates Macrophage Response in Microporous Annealed Particle (MAP) Scaffolds. Adv. Healthcare Mater. 2023, 12, e2300823 10.1002/adhm.202300823. PMC10592513 37165945

[ref41] Shangjing X.; Zhang L.; Phan N. V.; Carmichael S. T.; Segura T. Reactive astrocyte derived extracellular vesicles promote functional repair post stroke. bioRxiv 2022, 2022 10.1101/2022.09.06.506818.

[ref42] Ataie Z.; Kheirabadi S.; Zhang J. W.; Kedzierski A.; Petrosky C.; Jiang R.; Vollberg C.; Sheikhi A. Nanoengineered Granular Hydrogel Bioinks with Preserved Interconnected Microporosity for Extrusion Bioprinting (Small 37/2022). Small 2022, 18 (37), 2270196 10.1002/smll.202270196. 35922399

[ref43] Ding A.; Jeon O.; Cleveland D.; Gasvoda K. L.; Wells D.; Lee S. J.; Alsberg E. Jammed Micro-Flake Hydrogel for Four-Dimensional Living Cell Bioprinting. Adv. Mater. 2022, 34 (15), 2109394 10.1002/adma.202109394. PMC9012690 35065000

[ref44] Yuan Z.; Wan Z.; Tian Z.; Han Y.; Huang X.; Feng Y.; Xie W.; Duan X.; Huang S.; Liu X.; Huang J. In situ fused granular hydrogels with ultrastretchability, strong adhesion, and mutli-bioactivities for efficient chronic wound care. Chem. Eng. J. 2022, 450, 138076 10.1016/j.cej.2022.138076.

[ref45] Mendes B. B.; Daly A. C.; Reis R. L.; Domingues R. M. A.; Gomes M. E.; Burdick J. A. Injectable hyaluronic acid and platelet lysate-derived granular hydrogels for biomedical applications. Acta Biomater. 2021, 119, 101–113. 10.1016/j.actbio.2020.10.040. 33130309

[ref46] Zhang K.; Zhao W.; Fang H.; Chen X.; Hong Y.; Yin J.; Wang C. Low-fouling granular hydrogel for efficient preparation and delivery of stem cell spheroids towards wound treatment. Composites, Part B 2022, 246, 110239 10.1016/j.compositesb.2022.110239.

[ref47] Widener A. E.; Roberts A.; Phelps E. A. Single versus dual microgel species for forming guest-host microporous annealed particle PEG-MAL hydrogel. J. Biomed. Mater. Res., Part A 2023, 111, 1379–1389. 10.1002/jbm.a.37540. PMC10909382 37010360

[ref48] Widener A. E.; Bhatta M.; Angelini T. E.; Phelps E. A. Guest-host interlinked PEG-MAL granular hydrogels as an engineered cellular microenvironment. Biomater. Sci. 2021, 9 (7), 2480–2493. 10.1039/d0bm01499k. 33432940

[ref49] Widener A. E.; Duraivel S.; Angelini T. E.; Phelps E. A. Injectable Microporous Annealed Particle Hydrogel Based on Guest-Host-Interlinked Polyethylene Glycol Maleimide Microgels. Adv. NanoBiomed Res. 2022, 2 (10), 2200030 10.1002/anbr.202200030. 36419640 PMC9678130

[ref50] Trappmann B.; Baker B. M.; Polacheck W. J.; Choi C. K.; Burdick J. A.; Chen C. S. Matrix degradability controls multicellularity of 3D cell migration. Nat. Commun. 2017, 8 (1), 371 10.1038/s41467-017-00418-6. 28851858 PMC5575316

[ref51] van Dijk-Wolthuis W.; Franssen O.; Talsma H.; Van Steenbergen M.; Kettenes-Van Den Bosch J.; Hennink W. Synthesis, characterization, and polymerization of glycidyl methacrylate derivatized dextran. Macromolecules 1995, 28 (18), 6317–6322. 10.1021/ma00122a044.

[ref52] Ying W.; Cheruku P. S.; Bazer F. W.; Safe S. H.; Zhou B. Investigation of macrophage polarization using bone marrow derived macrophages. J. Visualized Exp. 2013, 76, e50323 10.3791/50323-v. PMC3728835 23851980

[ref53] Livak K. J.; Schmittgen T. D. Analysis of Relative Gene Expression Data Using Real-Time Quantitative PCR and the 2−ΔΔCT Method. Methods 2001, 25 (4), 402–408. 10.1006/meth.2001.1262. 11846609

[ref54] Seidi F.; Jin Y.; Xiao H. Polycyclodextrins: synthesis, functionalization, and applications. Carbohydr. Polym. 2020, 242, 116277 10.1016/j.carbpol.2020.116277. 32564845

[ref55] Daly A. C.; Riley L.; Segura T.; Burdick J. A. Hydrogel microparticles for biomedical applications. Nat. Rev. Mater. 2020, 5 (1), 20–43. 10.1038/s41578-019-0148-6. 34123409 PMC8191408

[ref56] Muir V. G.; Qazi T. H.; Weintraub S.; Torres Maldonado B. O.; Arratia P. E.; Burdick J. A. Sticking Together: Injectable Granular Hydrogels with Increased Functionality via Dynamic Covalent Inter-Particle Crosslinking. Small 2022, 18 (36), e2201115 10.1002/smll.202201115. 35315233 PMC9463088

[ref57] Mitchell M. J.; Billingsley M. M.; Haley R. M.; Wechsler M. E.; Peppas N. A.; Langer R. Engineering precision nanoparticles for drug delivery. Nat. Rev. Drug Discovery 2021, 20 (2), 101–124. 10.1038/s41573-020-0090-8. 33277608 PMC7717100

[ref58] Rodell C. B.; Ahmed M. S.; Garris C. S.; Pittet M. J.; Weissleder R. Development of Adamantane-Conjugated TLR7/8 Agonists for Supramolecular Delivery and Cancer Immunotherapy. Theranostics 2019, 9 (26), 8426–8436. 10.7150/thno.35434. 31879528 PMC6924259

[ref59] Kumar V. A.; Shi S.; Wang B. K.; Li I. C.; Jalan A. A.; Sarkar B.; Wickremasinghe N. C.; Hartgerink J. D. Drug-triggered and cross-linked self-assembling nanofibrous hydrogels. J. Am. Chem. Soc. 2015, 137 (14), 4823–4830. 10.1021/jacs.5b01549. 25831137 PMC4624388

[ref60] Lurier E. B.; Nash V. A.; Abee H. S.; Wissing T. B.; Bouten C. V. C.; Smits A.; Spiller K. L. Imparting Immunomodulatory Activity to Scaffolds via Biotin-Avidin Interactions. ACS Biomater. Sci. Eng. 2021, 7 (12), 5611–5621. 10.1021/acsbiomaterials.1c01190. 34767332

[ref61] Medina J. D.; Barber G. F.; Coronel M. M.; Hunckler M. D.; Linderman S. W.; Quizon M. J.; Ulker V.; Yolcu E. S.; Shirwan H.; García A. J. A hydrogel platform for co-delivery of immunomodulatory proteins for pancreatic islet allografts. J. Biomed. Mater. Res., Part A 2022, 110 (11), 1728–1737. 10.1002/jbm.a.37429. PMC12409600 35841329

[ref62] Kitagishi H.; Jiromaru M.; Hasegawa N. Intracellular Delivery of Adamantane-Tagged Small Molecule, Proteins, and Liposomes Using an Octaarginine-Conjugated β-Cyclodextrin. ACS Appl. Bio Mater. 2020, 3 (8), 4902–4911. 10.1021/acsabm.0c00421. 35021734

[ref63] Hashidzume A.; Harada A. Recognition of polymer side chains by cyclodextrins. Polym. Chem. 2011, 2 (10), 2146–2154. 10.1039/c1py00162k.

[ref64] Liu Y.; Liu Y.; Guo R. Insights into cyclodextrin-modulated interactions between protein and surfactant at specific and nonspecific binding stages. J. Colloid Interface Sci. 2010, 351 (1), 180–189. 10.1016/j.jcis.2010.07.032. 20701921

[ref65] Fu A. S.; Thatiparti T. R.; Saidel G. M.; von Recum H. A. Experimental studies and modeling of drug release from a tunable affinity-based drug delivery platform. Ann. Biomed. Eng. 2011, 39 (9), 2466–2475. 10.1007/s10439-011-0336-z. 21678091

[ref66] Fischer N. H.; Oliveira M. T.; Diness F. Chemical modification of proteins—challenges and trends at the start of the 2020s. Biomater. Sci. 2023, 11 (3), 719–748. 10.1039/D2BM01237E. 36519403

[ref67] Mansurov A.; Lauterbach A.; Budina E.; Alpar A. T.; Hubbell J. A.; Ishihara J. Immunoengineering approaches for cytokine therapy. Am. J. Physiol. 2021, 321 (2), C369–c383. 10.1152/ajpcell.00515.2020. 34232748

[ref68] Su X.; Yu Y.; Zhong Y.; Giannopoulou E. G.; Hu X.; Liu H.; Cross J. R.; Rätsch G.; Rice C. M.; Ivashkiv L. B. Interferon-γ regulates cellular metabolism and mRNA translation to potentiate macrophage activation. Nat. Immunol. 2015, 16 (8), 838–849. 10.1038/ni.3205. 26147685 PMC4509841

[ref69] Xian H.; Liu Y.; Rundberg Nilsson A.; Gatchalian R.; Crother T. R.; Tourtellotte W. G.; Zhang Y.; Aleman-Muench G. R.; Lewis G.; Chen W.; Kang S.; Luevanos M.; Trudler D.; Lipton S. A.; Soroosh P.; Teijaro J.; de la Torre J. C.; Arditi M.; Karin M.; Sanchez-Lopez E. Metformin inhibition of mitochondrial ATP and DNA synthesis abrogates NLRP3 inflammasome activation and pulmonary inflammation. Immunity 2021, 54 (7), 1463 10.1016/j.immuni.2021.05.004. 34115964 PMC8189765

[ref70] Spiller K. L.; Nassiri S.; Witherel C. E.; Anfang R. R.; Ng J.; Nakazawa K. R.; Yu T.; Vunjak-Novakovic G. Sequential delivery of immunomodulatory cytokines to facilitate the M1-to-M2 transition of macrophages and enhance vascularization of bone scaffolds. Biomaterials 2015, 37, 194–207. 10.1016/j.biomaterials.2014.10.017. 25453950 PMC4312192

[ref71] Bosurgi L.; Cao Y. G.; Cabeza-Cabrerizo M.; Tucci A.; Hughes L. D.; Kong Y.; Weinstein J. S.; Licona-Limon P.; Schmid E. T.; Pelorosso F.; Gagliani N.; Craft J. E.; Flavell R. A.; Ghosh S.; Rothlin C. V. Macrophage function in tissue repair and remodeling requires IL-4 or IL-13 with apoptotic cells. Science 2017, 356 (6342), 1072–1076. 10.1126/science.aai8132. 28495875 PMC5556699

[ref72] Noe J. T.; Rendon B. E.; Geller A. E.; Conroy L. R.; Morrissey S. M.; Young L. E. A.; Bruntz R. C.; Kim E. J.; Wise-Mitchell A.; Barbosa de Souza Rizzo M.; Relich E. R.; Baby B. V.; Johnson L. A.; Affronti H. C.; McMasters K. M.; Clem B. F.; Gentry M. S.; Yan J.; Wellen K. E.; Sun R. C.; Mitchell R. A. Lactate supports a metabolic-epigenetic link in macrophage polarization. Sci. Adv. 2021, 7 (46), eabi8602 10.1126/sciadv.abi8602. 34767443 PMC8589316

[ref73] Baseler W. A.; Davies L. C.; Quigley L.; Ridnour L. A.; Weiss J. M.; Hussain S. P.; Wink D. A.; McVicar D. W. Autocrine IL-10 functions as a rheostat for M1 macrophage glycolytic commitment by tuning nitric oxide production. Redox Biol. 2016, 10, 12–23. 10.1016/j.redox.2016.09.005. 27676159 PMC5037266

[ref74] Ip W. K. E.; Hoshi N.; Shouval D. S.; Snapper S.; Medzhitov R. Anti-inflammatory effect of IL-10 mediated by metabolic reprogramming of macrophages. Science 2017, 356 (6337), 513–519. 10.1126/science.aal3535. 28473584 PMC6260791

[ref75] Saxton R. A.; Tsutsumi N.; Su L. L.; Abhiraman G. C.; Mohan K.; Henneberg L. T.; Aduri N. G.; Gati C.; Garcia K. C. Structure-based decoupling of the pro- and anti-inflammatory functions of interleukin-10. Science 2021, 371 (6535), eabc8433 10.1126/science.abc8433. 33737461 PMC9132103

[ref76] Shouval D. S.; Biswas A.; Goettel J. A.; McCann K.; Conaway E.; Redhu N. S.; Mascanfroni I. D.; Al Adham Z.; Lavoie S.; Ibourk M.; Nguyen D. D.; Samsom J. N.; Escher J. C.; Somech R.; Weiss B.; Beier R.; Conklin L. S.; Ebens C. L.; Santos F. G.; Ferreira A. R.; Sherlock M.; Bhan A. K.; Muller W.; Mora J. R.; Quintana F. J.; Klein C.; Muise A. M.; Horwitz B. H.; Snapper S. B. Interleukin-10 receptor signaling in innate immune cells regulates mucosal immune tolerance and anti-inflammatory macrophage function. Immunity 2014, 40 (5), 706–719. 10.1016/j.immuni.2014.03.011. 24792912 PMC4513358

[ref77] Mumm J. B.; Emmerich J.; Zhang X.; Chan I.; Wu L.; Mauze S.; Blaisdell S.; Basham B.; Dai J.; Grein J.; Sheppard C.; Hong K.; Cutler C.; Turner S.; LaFace D.; Kleinschek M.; Judo M.; Ayanoglu G.; Langowski J.; Gu D.; Paporello B.; Murphy E.; Sriram V.; Naravula S.; Desai B.; Medicherla S.; Seghezzi W.; McClanahan T.; Cannon-Carlson S.; Beebe A. M.; Oft M. IL-10 Elicits IFNγ-Dependent Tumor Immune Surveillance. Cancer Cell 2011, 20 (6), 781–796. 10.1016/j.ccr.2011.11.003. 22172723

[ref78] Tilg H.; van Montfrans C.; van den Ende A.; Kaser A.; van Deventer S. J.; Schreiber S.; Gregor M.; Ludwiczek O.; Rutgeerts P.; Gasche C.; Koningsberger J. C.; Abreu L.; Kuhn I.; Cohard M.; LeBeaut A.; Grint P.; Weiss G. Treatment of Crohn’s disease with recombinant human interleukin 10 induces the proinflammatory cytokine interferon gamma. Gut 2002, 50 (2), 191–195. 10.1136/gut.50.2.191. 11788558 PMC1773093

[ref79] Spiller K. L.; Wrona E. A.; Romero-Torres S.; Pallotta I.; Graney P. L.; Witherel C. E.; Panicker L. M.; Feldman R. A.; Urbanska A. M.; Santambrogio L.; Vunjak-Novakovic G.; Freytes D. O. Differential gene expression in human, murine, and cell line-derived macrophages upon polarization. Exp. Cell Res. 2016, 347 (1), 1–13. 10.1016/j.yexcr.2015.10.017. 26500109

