# Supplementary material for: Injectable Granular Hydrogels Enable Avidity-Controlled Biotherapeutic Delivery
Source: ACS Biomater Sci Eng. 2024 Feb 15;10(3):1577–88. doi: 10.1021/acsbiomaterials.3c01906 (PMC10934254; doi:10.1021/acsbiomaterials.3c01906)
Supplement: Supplementary file 1 — ab3c01906_si_001.pdf [file ab3c01906_si_001.pdf]

Supporting Information

Injectable Granular Hydrogels Enable Avidity-Controlled Biotherapeutic Delivery

Arielle M. D'Elia[1], Olivia L. Jones[1], Gabriela Canziani[2], Biplab Sarkar[1], Irwin Chaiken[2], Christopher B. Rodell[1]*

[1]*School of Biomedical Engineering, Science and Health Systems, Drexel University, Philadelphia, PA, USA*

[2]*Department of Biochemistry and Molecular Biology, Drexel University College of Medicine, Philadelphia, PA, USA*

* Correspondence to: C.B. Rodell (christopher.b.rodell@drexel.edu)

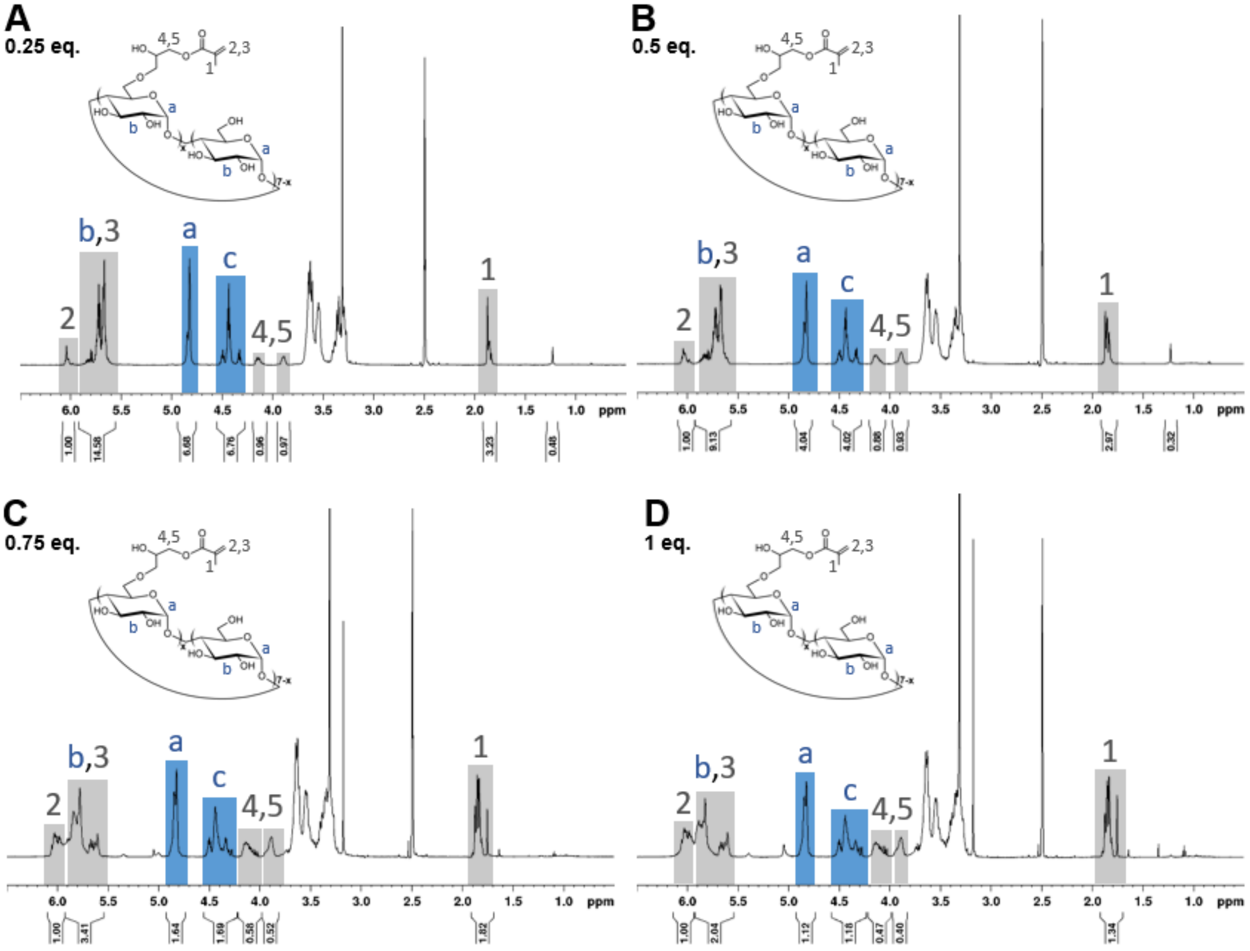

Figure S1. ^{1}H-NMR spectra of (A) 0.25 eq., (B) 0.5 eq., (C) 0.75 eq., and (D) 1 eq. GMA per glucose repeat unit. The degree of substitution is determined by the ratio of the methyl peak (3H, ca. 1.9 ppm) relative to the position 1 anomeric proton (7H, ca. 4.8 ppm).

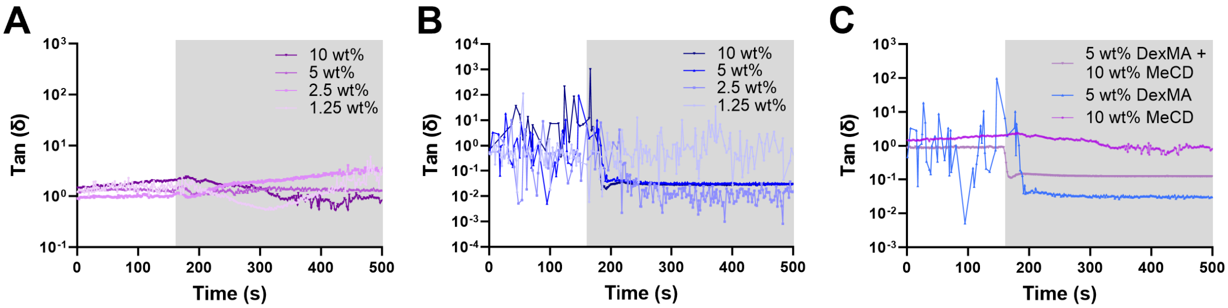

Figure S2. Viscoelastic properties are tuned via DexMA incorporation. Representative oscillatory time sweeps (1 Hz, 1.0% strain) of photo-crosslinking with UV exposure (10 mW/cm^2, 5 min as indicated by the shaded area) for MeCD (A), DexMA (B), and composite (C) hydrogels. A clear transition to a solid hydrogel (tan(δ)<1) was observed for DexMA at concentrations of 2.5 wt% or greater.

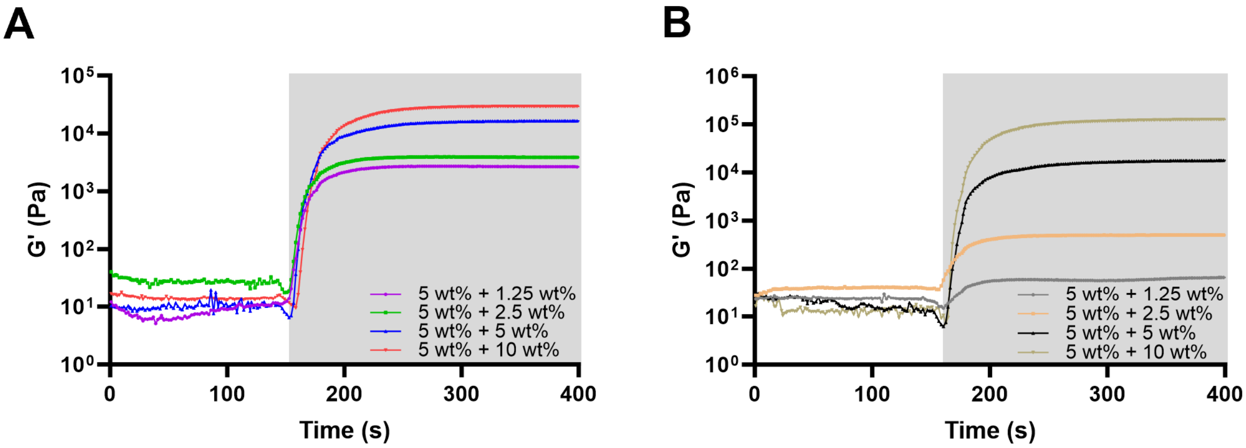

Figure S3. Rheological characterization of hydrogel formation. Representative oscillatory time sweeps of photo-crosslinking with UV exposure (10 mW/cm^2, 5 min as indicated by the shaded area) showing shear storage moduli (G') of 5%$_{w/v}$ DexMA with increasing MeCD (A) and 5%$_{w/v}$ MeCD with increasing DexMA (B), each at 1 Hz, 1.0% strain.

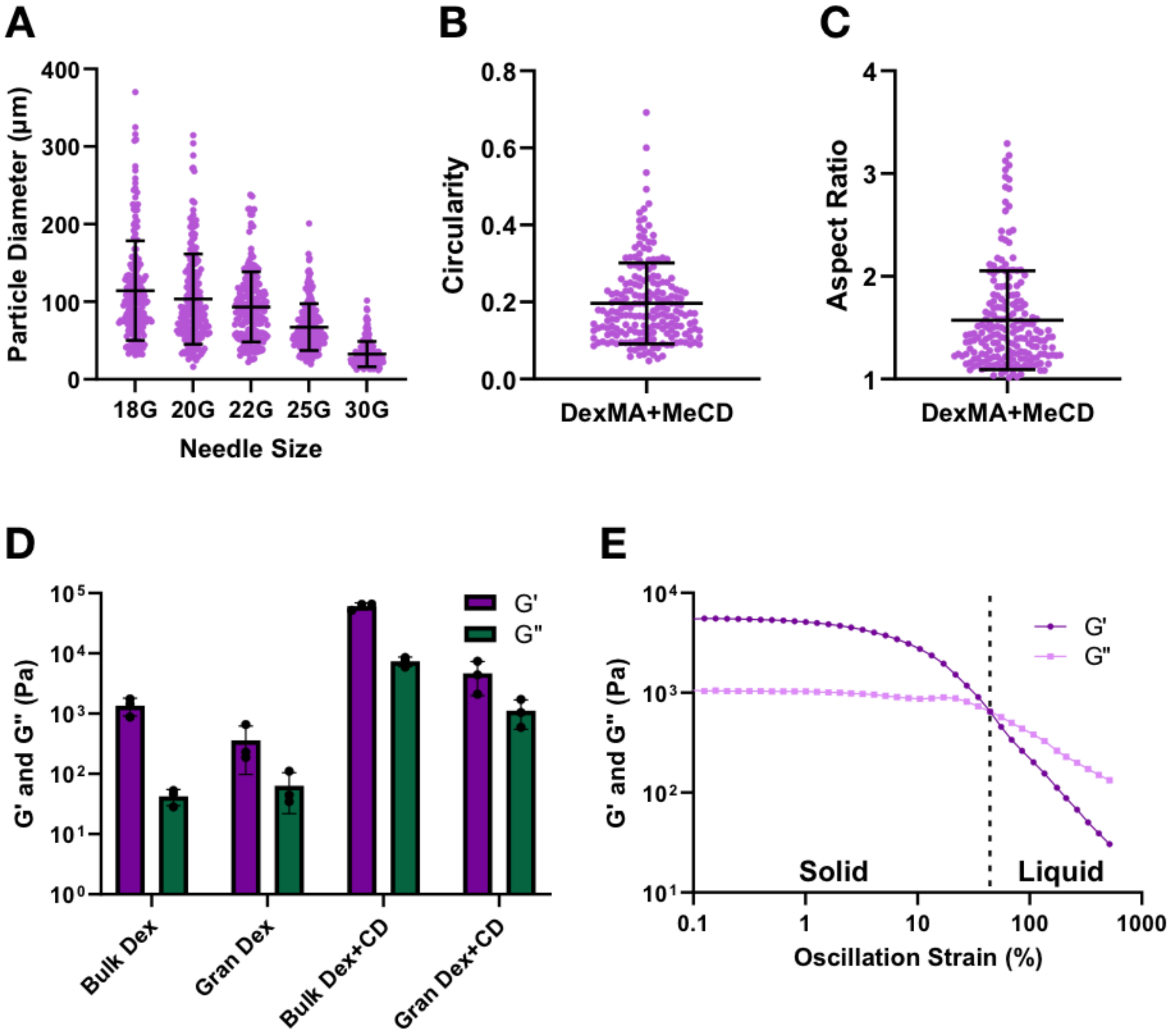

Figure S4. Granular hydrogel formation and mechanical properties. (A) Particle diameter throughout the extrusion process of 5%$_{w/v}$ DexMA + 10%$_{w/v}$ MeCD gels; mean ± SD; n=200 particles. (B, C) Corresponding quantification of particulate circularity and aspect ratio for final microgels obtained after 30G needle extrusion; mean ± SD, n=200. (D) Storage (G', purple) and loss (G", green) moduli comparison of granular and bulk hydrogels composed of 5%$_{w/v}$ DexMA and 5%$_{w/v}$ DexMA + 10%$_{w/v}$ MeCD (1.0% strain, 1 Hz); mean ± SD, n=3. (E) Strain amplitude sweep of 5%$_{w/v}$ DexMA + 10%$_{w/v}$ MeCD granular hydrogels (1 Hz); G' (dark purple, circle), G" (light purple, circle).

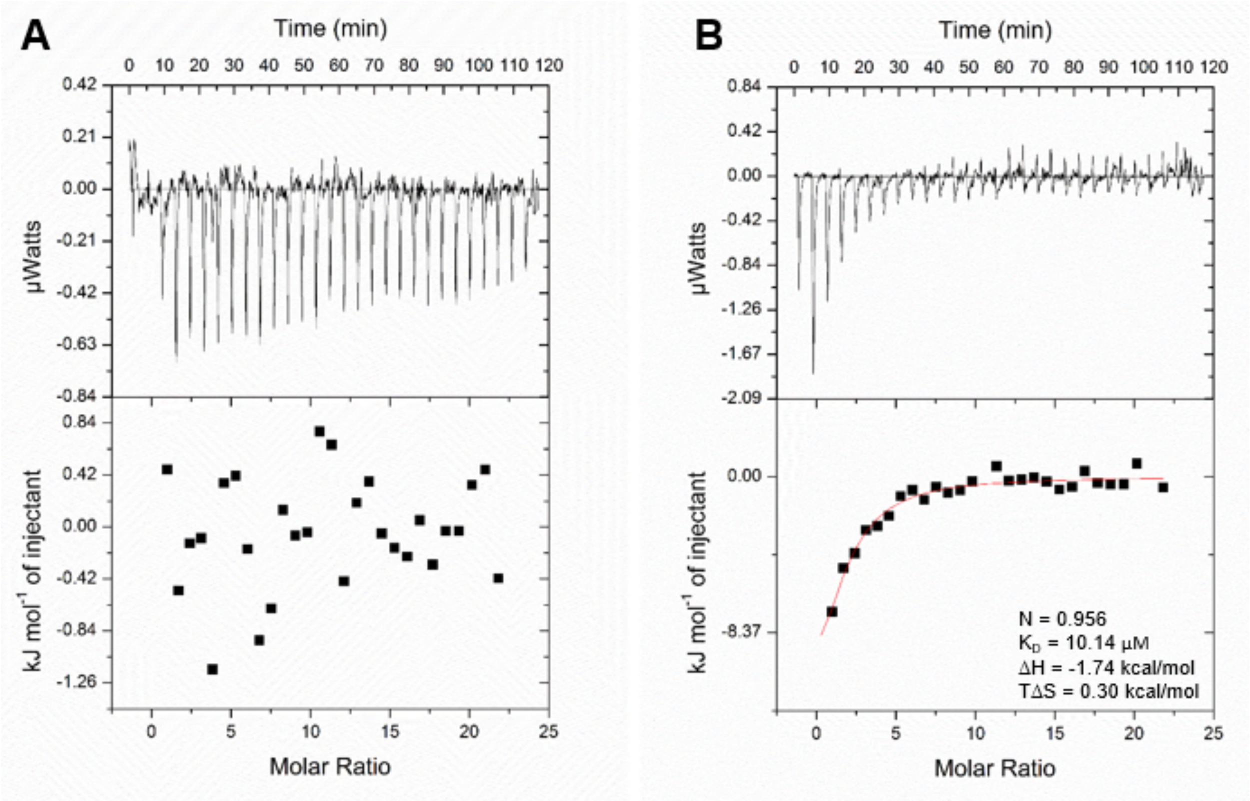

Figure S5. Isothermal titration calorimetry (ITC) thermodynamic evaluation. Raw thermograms and binding isotherms of controls, including unmodified BSA (A) and soluble Ad (B) to CD.

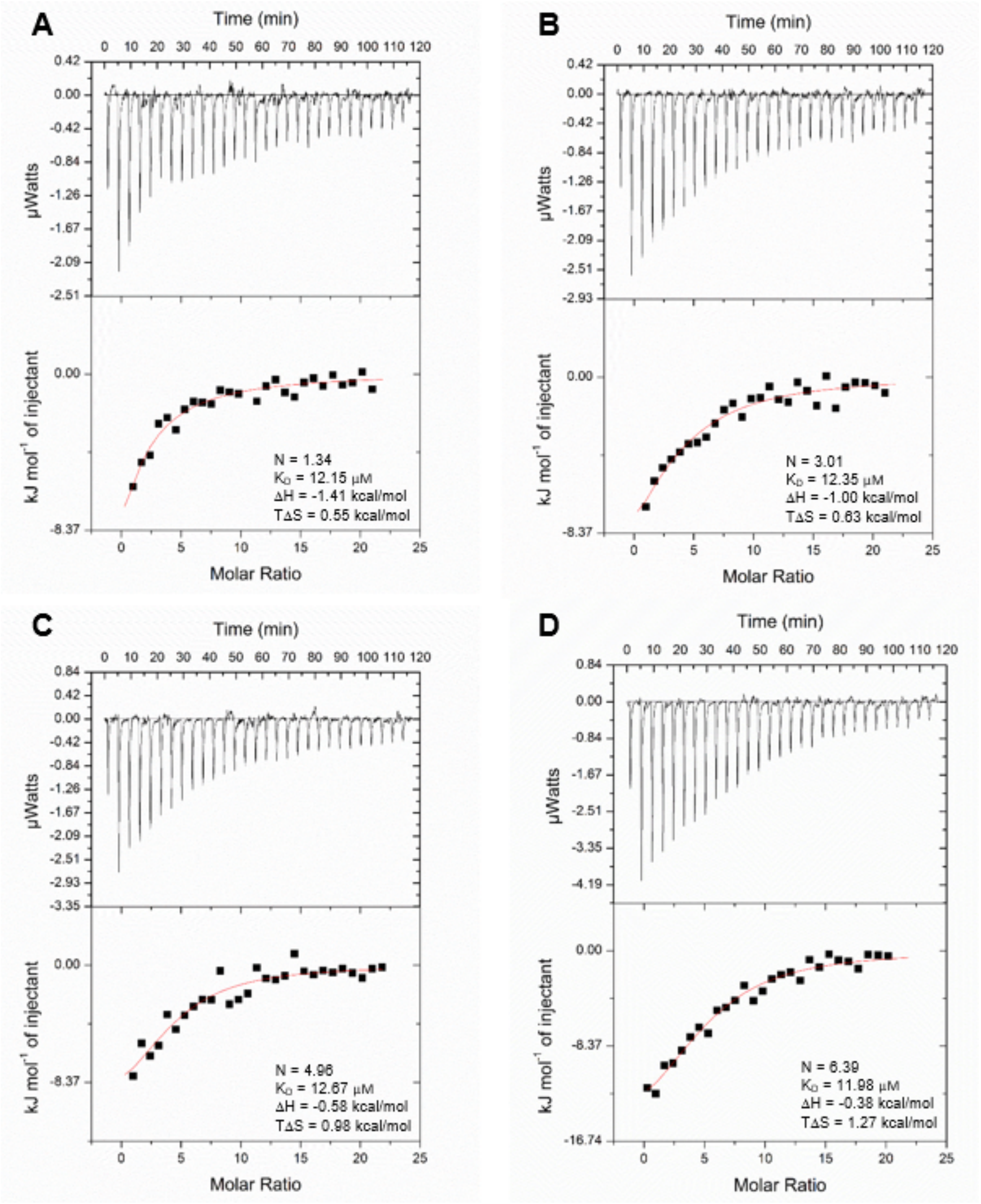

Figure S6. Isothermal titration calorimetry (ITC) thermodynamic evaluation. Raw thermograms and binding isotherms of Ad-BSA conjugates (A-D).

Equivalents adamantane-BSA	K_D (nM)	Residuals square (43570 average data points)
1.25	356	0.89
2.50	253	0.85
5.00	207	0.69
10.00	164	0.51

Table 1. Dissociation constant of Ad-BSA, determined by SPR. The equilibrium dissociation constant (K_D) reflects the apparent avidity of multimeric adamantane binding to surface-coupled β-cyclodextrin. The K_D shifts to lower (tighter) nM values with increasing adamantane content of BSA. Ad-BSA conjugates binding to β-cyclodextrin was assessed in individual kinetic titration cycles and the resulting data fit to a 1:1 interaction model to determine the avidity of multimeric binding.
